# Observations of Closed Magnetic Flux Embedded in the Lobes During Periods of Northward IMF

**DOI:** 10.1029/2021JA029281

**Published:** 2021-06-01

**Authors:** L. J. Fryer, R. C. Fear, J. C. Coxon, I. L. Gingell

**Affiliations:** ^1^ School of Physics & Astronomy University of Southampton Southampton UK

## Abstract

The high latitude, lobe regions of the magnetosphere are often assumed to contain cool, low energy plasma populations. However, during periods of northward Interplanetary Magnetic Field, energetic plasma populations have occasionally been observed. We present three cases when Cluster observed uncharacteristically “hot” plasma populations in the lobe. For two of the three events, we present simultaneous observations of the plasma sheet observed by Double Star. The similarity between the plasma in the lobe and the plasma sheet suggests that the mechanism that produces plasma at high latitudes is likely to be tail reconnection, resulting in a trapped “wedge” of closed flux about the noon‐midnight meridian. Complementary images from Imager for Magnetopause to Aurora Global Exploration and DMSP/Special Sensor Ultraviolet Spectrographic Imager show that transpolar arcs, which form in each event in at least one hemisphere, directly intersect the footprint of the Cluster spacecraft in all three events. The intersection of the Cluster footprint with the transpolar arcs is synchronous with the observation of the energetic plasma populations in the lobe. This further supports the conclusion that it is likely this energetic plasma observed in the high latitude lobe regions of magnetosphere is on closed field lines.

## Key Points

- Cluster observations of hot plasma in the lobe are directly comparable to simultaneous observations of the plasma sheet by Double Star
- Plasma observations suggest that tail reconnection is the cause for the presence of energetic plasma in the high latitude magnetosphere
- Imaging spacecraft support previous findings which show an association between transpolar arcs and energetic plasma observed in the lobe

## 1 Introduction

The coupling between the Interplanetary Magnetic Field (IMF) and the magnetosphere has been extensively studied over the last few decades, but there are still many unanswered questions with regard to how the magnetosphere responds to different IMF conditions, particularly when the IMF is northward. Under southward IMF, reconnection occurs on the dayside and the cyclic process proposed by Dungey (1961) is widely accepted. However, under northward IMF, reconnection occurs at higher latitudes and the “traditional” convection process changes (Cowley, 1981; Crooker, 1992; Cumnock et al., 1995; Dungey, 1963; Fear, 2021; Russell, 1972). In particular, the configuration and composition of the magnetospheric lobes under northward IMF are not well understood and have yet to be extensively studied.

The lobes are typically described as having cool and often low‐density plasma populations; hence, hot plasma observations are unexpected in this region of the magnetosphere (e.g., Svenes et al., 2008). Despite this, there have been a number of studies reporting energetic plasma populations in the lobes during northward IMF conditions (Fear et al., 2014; Huang et al., 1987, 1989; Shi et al., 2013). This hot plasma has also been shown to coincide with observations of transpolar arcs (TPAs) (Fear et al., 2014; Huang et al., 1989; Mailyan et al., 2015; Reidy et al., 2018), which are structures observed poleward of the main auroral oval, typically bisecting the polar region during periods of northward IMF. Cooler “Polar Cap Ion Beams” have also been observed at lower altitudes above polar cap auroras (e.g., Maggiolo et al., 2012). TPAs can last on timescales from minutes to hours (Kullen et al., 2002). Current research is still ongoing to answer the question of how these TPAs are formed (See review by Hosokawa et al., 2020).

The origin of the observations of energetic plasma observed in the lobes has long been debated (Huang et al., 1987). Different mechanisms can be broadly separated by the field line topology surrounding these plasma populations and whether it is present on open or closed field lines. In this study we look at two specific, contrasting theories, which describe mechanisms which could lead to the presence of “hot” plasma embedded in the lobes (Milan et al., 2005; Shi et al., 2013).

Milan et al. (2005) proposed a mechanism for the formation of TPAs which also explains the presence of energetic plasma at high latitudes. This mechanism can be summarized as being a result of the occurrence of tail reconnection that is observed under northward IMF conditions (Grocott et al., 2003, 2004). When tail reconnection occurs, cold lobe plasma can become trapped on newly closed magnetic field lines. The newly closed field lines contract and consequently heat the enclosed plasma. This process is known to form the plasma sheet population under southward IMF conditions, however, Milan et al. (2005) argue that under northward IMF conditions, the contraction and return flow (to the day side) of the closed field lines can be frustrated in the midnight sector. This causes a build up of closed magnetic flux to occur forming a “wedge” which emerges from the plasma sheet (Fear et al., 2015). This theory is supported by statistical analysis of the formation of transpolar arcs (Fear & Milan, 2012), and has been used to explain a case study of uncharacteristically hot plasma embedded in the lobe (Fear et al., 2014). In the study by Fear et al. (2014), the observations within the lobe were compared to average plasma sheet characteristics during northward IMF conditions from Walsh et al. (2013). Plasma sheet characteristics can vary and hence we would not expect all northward IMF conditions to show the same plasma sheet characteristics (Maggiolo & Kistler, 2014). A more reliable approach which enables a direct comparison to be made is to use simultaneous in‐situ observations of plasma populations observed both at high latitude and those observed within the plasma sheet, which is the approach taken within this study.

A second possible mechanism is that “hot” plasma is seen in the lobe due to direct entry from the solar wind on open magnetic field lines, as described by Shi et al. (2013) and subsequently reported by Mailyan et al. (2015) and Gou et al. (2016). Shi et al. (2013) interpreted this direct entry of solar wind plasma into the magnetosphere as being due to high‐latitude reconnection of open lobe field lines during Northward IMF, although the authors were unable to exclude completely impulsive penetration as a plausible entry mechanism.

**Figure 1:**
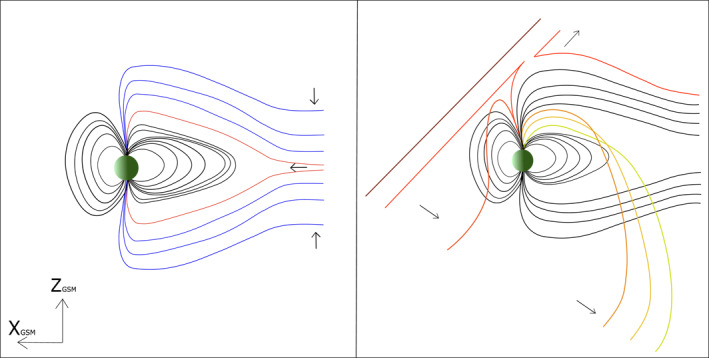
This schematic represents the difference between the two proposed mechanisms which explain how hot energetic plasma can be observed in the typically cool lobes of the magnetosphere. The left diagram represents the topology of the magnetotail during tail reconnection during non‐substorm intervals as described by Milan et al. (2005). The blue lines represent lobe field lines which are open and will ultimately undergo tail reconnection far downtail. The field lines that have reconnected, represented with red field lines, contract earthward to form a “wedge” of closed flux about the noon‐midnight meridian on the nightside. A similar schematic was produced by Fear et al. (2015) (Figure 3) which details the expected configuration of the magnetosphere once tail reconnection has occurred and a build up of flux is present at a discrete local time in the lobe. In contrast, the diagram on the right represents the expected topology when high‐latitude reconnection occurs with the lobe during northward IMF, a direct result of the mechanism proposed by Shi et al. (2013). The red field lines show high latitude reconnection. They are then subsequently convected anti‐sunward, as expected for typical northward IMF conditions, stretching the open field lines as they are dragged anti‐sunward (orange to yellow field lines) with the propagating IMF and are progressively stretched.

Both the Milan et al. (2005) and Shi et al. (2013) models are reliant on a Northward orientated IMF, so cannot be differentiated using IMF dependency alone. A key testable difference between the mechanisms is based on the stretching/contraction of magnetic field lines, which is illustrated in Figure 1. In the Milan et al. (2005) mechanism, the hot plasma population is found on field lines that have been recently closed by magnetotail reconnection, and have therefore contracted to some degree from their pre‐reconnection stretched lobe configuration (Figure 1, left). On the other hand, since the Shi et al. (2013) mechanism is based on high latitude magnetopause reconnection, and the plasma signatures in question are observed significantly tailward of the cusp (e.g., at *X* = −8 R_E_ in the example shown by Shi et al., 2013), we would expect an initial contraction of the reconnected field line earthward; this would be followed by a progressive stretching of field lines as they convect around to the nightside due to reverse convection that occurs under northward IMF (Cowley, 1981; Crooker, 1992; Dungey, 1963; Haaland et al., 2008). This means that in the Milan et al. (2005) mechanism, closed field lines are progressively more contracted at lower latitudes, and we would expect them to be associated with progressively hotter plasma populations as a result, whereas in the Shi et al. (2013) mechanism, field lines which are at lower latitudes, closer to the plasma sheet, should be more stretched than those which are at higher latitude, resulting in cooler plasma distributions at lower latitudes.

Another distinction between the Milan et al. (2005) and Shi et al. (2013) mechanisms is that the plasma should bear similarities to the plasma sheet or solar wind respectively. Shi et al. (2013) performed a statistical analysis and found that higher plasma densities in these lobe events corresponded to higher solar wind densities. On the other hand, Fear et al. (2014) investigated energetic plasma seen by Cluster and concluded that this hot plasma was similar in distribution and temperature to the values commonly found in the (relatively cool) plasma sheet during northward IMF conditions (Walsh et al., 2013). Following on from this, Fear et al. (2014) studied electron pitch angle distributions, which showed evidence of a double loss cone. This finding, in conjunction with the similarity of the temperature and density to that of the plasma sheet, supported the theory that the origin of the plasma observed in the lobe was not likely to be from direct solar wind entry (which require an open topology), but can be explained well by tail reconnection and hence form due to the closure of magnetic field lines in the lobe. This conclusion was further supported by the observation of a TPA which is prominent throughout the period of interest.

Following on from the investigation undertaken by Fear et al. (2014), we provide further supporting observations from September 15, 2005 and investigate two other conjunctions for which the IMF is northward. We use direct, simultaneous observations of the plasma sheet and additionally utilize the separation of the Cluster tetrahedron in Event 2 to investigate the spatial structure of these plasma populations in more detail. We discuss the instrumentation used to probe the magnetosphere in Section 2, and provide quantitative analysis of plasma parameters and examine auroral images from over the polar regions in Section 3. In Section 4 we compare the results of the data analysis to current formation models of TPAs and determine that the hot plasma embedded in the lobe is likely to have formed on closed field lines. In particular we conclude that the similarity of the observations of the plasma sheet with that of the lobe plasma populations are consistent with the mechanism proposed by Milan et al. (2005), and not with direct entry from the solar wind (Shi et al., 2013). In all three events, we simultaneously observe evidence of a transpolar arc formation. We show for the first time that multiple hot plasma populations that are embedded in the lobes are not necessarily on adjacent closed field lines; as evidenced by Event 2 they can be separated in local time by open lobe field lines, corresponding to multiple arc structures. This study thus supports both the proposed model of TPA formation, and that the presence of energetic plasma in the lobe is a result of nightside tail reconnection. The latter process forms a wedge of trapped closed field lines surrounded by the typical open lobe field lines (Fear et al., 2015; Milan et al., 2005).

## 2 Instrumentation

We use multiple spacecraft missions to provide both image and particle data to probe different regions of the magnetosphere. Cluster, which was launched in 2000 into a polar orbit (Escoubet et al., 2001, 2013, 2015), provides information on the ion and electron populations through the Cluster Ion Spectrometer (CIS) (Dandouras et al., 2010; Reme et al., 1997) and Plasma Electron And Current Experiment (PEACE) (Fazakerley et al., 2010; Johnstone et al., 1997) instruments, respectively. The Fluxgate Magnetometer (FGM) is used to measure the local magnetic field (Balogh et al., 1997, 2001; Gloag et al., 2010). These instruments are situated on four separate spacecraft, which collectively make up the Cluster mission. The four spacecraft can be maneuvered to separate distances from tens to thousands of kilometers apart, depending on the regions of magnetospheric interest and mission phase (Escoubet et al., 2001, 2013, 2015).

Double Star (Escoubet et al., 2005; Liu et al., 2005), was launched in December 2003 to observe the magnetosphere simultaneously to Cluster. In this study, we use data from the equatorial Double Star spacecraft (TC‐1). Plasma data are provided by the PEACE instrument, which measures electron particle distributions in up to three dimensions (Fazakerley et al., 2005), and the Hot Ion Analyzer (HIA), which measures ion properties (Rème et al., 2005). The product of this instrument are analogous to those provided by the HIA sensor that constitutes part of Cluster's CIS instrument.

The Special Sensor Ultraviolet Spectrographic Imager (SSUSI), which was launched on the DMSP 5D‐F16 spacecraft in 2003, is a scanning instrument that provides images of the aurora over the poles. SSUSI provides us with low altitude, high resolution images of the aurora in the polar regions. Due to the fact that the DMSP spacecraft are in low Earth orbit, SSUSI can observe both the North and South Poles multiple times a day (Paxton et al., 1992).

The Imager for Magnetopause to Aurora Global Exploration (IMAGE) spacecraft housed a Far Ultraviolet (FUV) Wide‐band Imaging Camera (WIC) designed to observe the aurora at wavelengths between 140‐190 nm (Mende et al., 2000). WIC had capabilities of resolving aurora down to scales of 2° latitude. We use data from WIC to provide global images of the southern hemisphere (The northern hemisphere can also be observed but the spacecraft was not located in this region for any of the events discussed in this study). The OMNI data set is used to provide 1‐min resolution measurements of the IMF propagated to the nose of the bow‐shock (King & Papitashvili, 2005).

## 3 Observations

In this section, we provide a recap of the observation reported by Fear et al. (2014), provide new observations of the plasma sheet at that time, and introduce two further events which will allow us to probe the mechanism predictions discussed above. These three events were selected as times when a TPA was present in the survey by Fear and Milan (2012), and Cluster was situated in the lobe, but observed plasma uncharacteristic of the lobe. Table 1 shows the date on which each event occurred; we refer to the events by event number throughout the rest of this study.

**Table 1:** The Date and Time of Each Event

Event	Date	Time interval
Event 1	September 15, 2005	13:00–20:00 UT
Event 2	September 30, 2005	15:00–22:00 UT
Event 3	September 11, 2003	04:00–09:00 UT

### 3.1 Event 1

**Figure 2:**
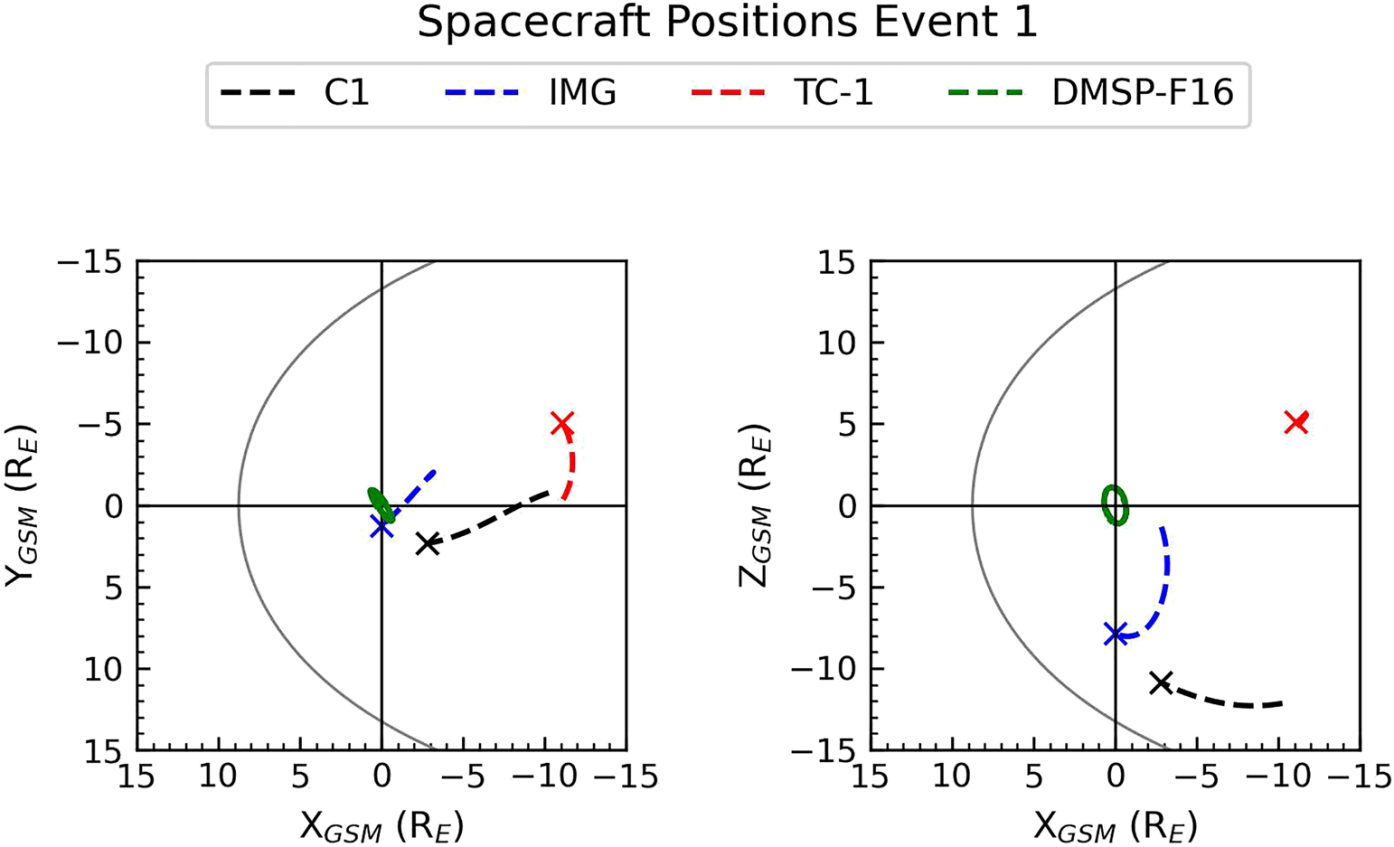
Orbit trajectories of the spacecraft used to investigate Event 1. The trajectories of Cluster 1, Imager for Magnetopause to Aurora Global Exploration, TC‐1, and DMSP‐F16 can be seen in black, blue, red and green respectively. The asterisk marks the spacecraft location at the end of the period of interest at 20:00 UT. The average magnetopause position is modeled (Shue et al., 1998) and shown in gray.

On the September 15, 2005 between 13:00 UT and 20:00 UT, the Cluster spacecraft were located in the Southern Hemisphere lobe region. Figure 2 shows the locations of Cluster, IMAGE, TC‐1, and DMSP‐F16 spacecraft in GSM coordinates. From this figure it can be seen that TC‐1 is located at lower latitude than Cluster and hence provides us with simultaneous plasma sheet observations; IMAGE and SSUSI (on board DMSP‐F16) provide high and low‐altitude observations of the poles respectively.

**Figure 3:**
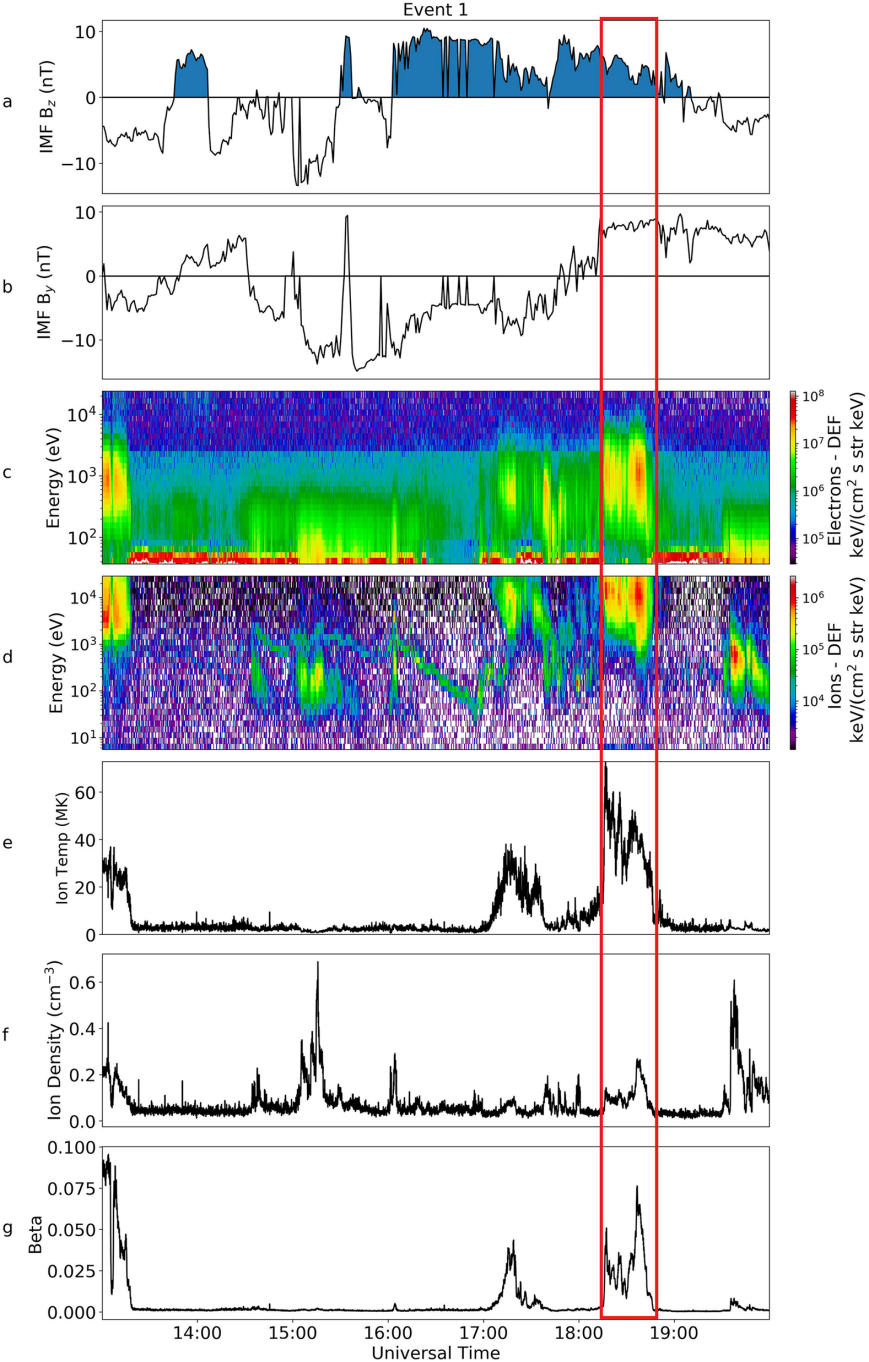
Magnetic and particle data from OMNI and Cluster 1 for Event 1. Panel a shows the B_*z*_ component of the Interplanetary Magnetic Field (IMF) taken from the OMNI data set. The blue shading marks when the IMF was northward (*B*_*z*_ > 0). Panel b shows the IMF B_*y*_ component, again from OMNI. The next four panels all show data from Cluster 1. Panel c shows a spectrogram of the energy of the electrons from the Plasma Electron And Current Experiment instrument and d, the differential energy flux of ions from the Hot Ion Analyzer instrument. The final three panels, e–g, show the temperature, density, and beta parameter respectively. The spacecraft potential has been plotted over the electron spectrogram in white.

**Figure 4:**
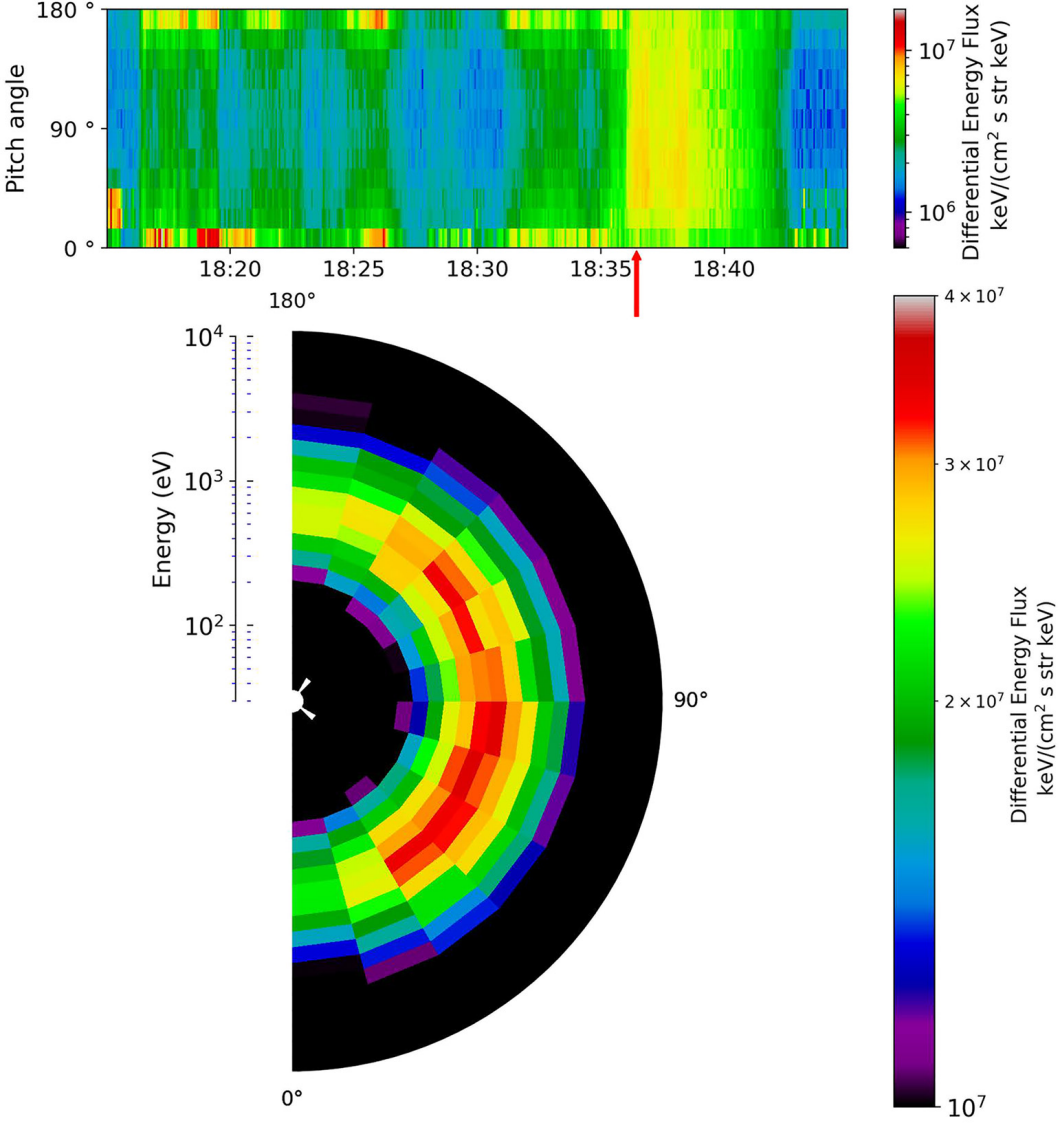
The electron pitch angle and energy distribution observed by Cluster 1 for Event 1. The top panel shows the differential energy flux distribution of electrons with respect to pitch angle as a function of time between 18:15 UT and 18:45 UT (region within the red box in Figure 3). The lower plot shows the energy with respect to pitch angle for an average of five time stamps centered about 18:36 UT (indicated by the red arrow).

Figure 3 shows a summary of the key observations from Cluster 1 for Event 1, as reported by Fear et al. (2014). Panels a and b show the IMF B_*z*_ and B_*y*_ components, c and d are the electron and ion spectrograms, panels e and f show the measured temperature and density of the ions and panel g is the plasma beta. A little while after the northward turning of the IMF at 16:00 UT, Cluster observed electron and ion populations with energies centered at 10^3^ and 10^4^ eV respectively. Ion temperatures for this event also peaked between 40 and 60 MK. Fear et al. (2014) examined the electron pitch angle distribution (PAD), which is also plotted here in Figure 4. The top panel of this figure shows a pitch angle spectrogram of the electrons over a period of 30 min from 18:15 UT–18:45 UT (region within the red box in Figure 3). For the majority of this event a bi‐directional distribution was observed, peaking at 0° and 180°. However, there were also periods when the pitch angle distribution peaked closer to 90°, indicative of a double loss cone and hence suggestive of the spacecraft being on closed field lines. An example is shown in the lower panel of Figure 4, which shows an average of the pitch angle distribution taken over five spins at 18:36 UT, which corresponds to the position at the red arrow in the top panel.

**Figure 5:**
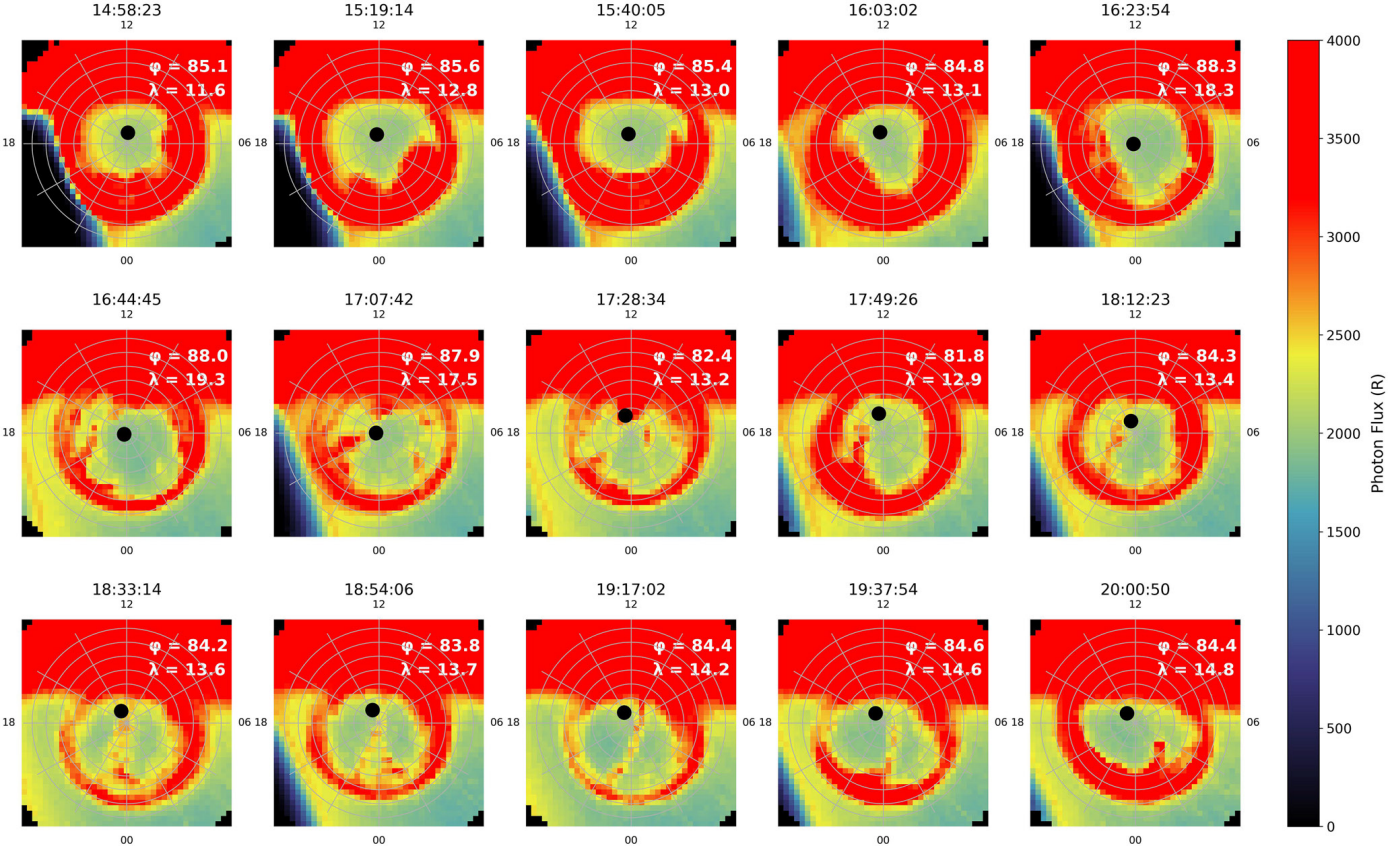
Imager for Magnetopause to Aurora Global Exploration Far Ultraviolet wide‐band imaging camera observations of the Southern Hemisphere for Event 1, plotted in Altitude Adjusted Corrected Geomagnetic (AACGM) magnetic latitude and magnetic local time. We have adopted the convention of plotting noon MLT at the top, and dusk at the left hence these southern hemisphere observations are shown as if viewed through the planet from the north. The panels shown correspond to the period that Cluster was in the lobe, including the period during which it observed the “hot” plasma. We present the data in intervals of 20 min starting with the time stamp 14:58 UT. The black circle represents the footprint position from Cluster 1 mapped to 120 km in altitude using the T96 model, also plotted in AACGM coordinates. The magnetic latitude and magnetic local time of Cluster 1 is noted in the top right corner of each panel. Between 17:07 UT and 19:17 UT the footprint can be seen to intersect with the transpolar arc as it moves across the polar cap.

Fear et al. (2014) used FUV images from the IMAGE spacecraft to identify a TPA which coincided with the measurements from Cluster. This correspondence was confirmed by the mapping of footprints (Tsyganenko, 1996) of the spacecraft to the IMAGE data in which the TPA was visible. This provided evidence that the plasma observed in the particle data was the same plasma population responsible for the TPA. IMAGE FUV auroral observations can be seen in Figure 5 (Data are provided by the Cluster Science Archive [CSA] and have been pre‐processed onto a 40 × 40 grid with 222 km spacing). The key observations are as follows: initially an oval brightening was observed on the duskside between 16 and 23 MLT at 16:23 UT (top row of Figure 5), which is just prior to the higher energy population being observed by Cluster (Figure 3c). By 16:44 UT, a distinct TPA was observed, which appeared to span across the entire polar cap. At 17:07 UT the footprint of the Cluster spacecraft, traced to 120 km altitude using the Tsyganenko 96 model (Tsyganenko, 1996), intersects with the arc, which moved dawnward toward to noon‐midnight meridian. The arc then appeared to fade for a short period around 17:28 UT. We observe the TPA moving duskward between 17:28 UT and 18:12 UT. By 18:33 UT there was a second intersection between the footprint and the TPA which corresponds to the next time at which Cluster observed “hot” plasma in the lobe regions. The TPA then remained underneath the footprint until 19:17 UT, at which point further dawnward motion occurs. The TPA was present in the IMAGE data until just after 20:00 UT. Fear et al. (2014) demonstrated that for the timestamps which show the direct intersection of the footprint and the TPA, energetic plasma was present at high latitudes in the lobe (as seen by Cluster).

**Figure 6:**
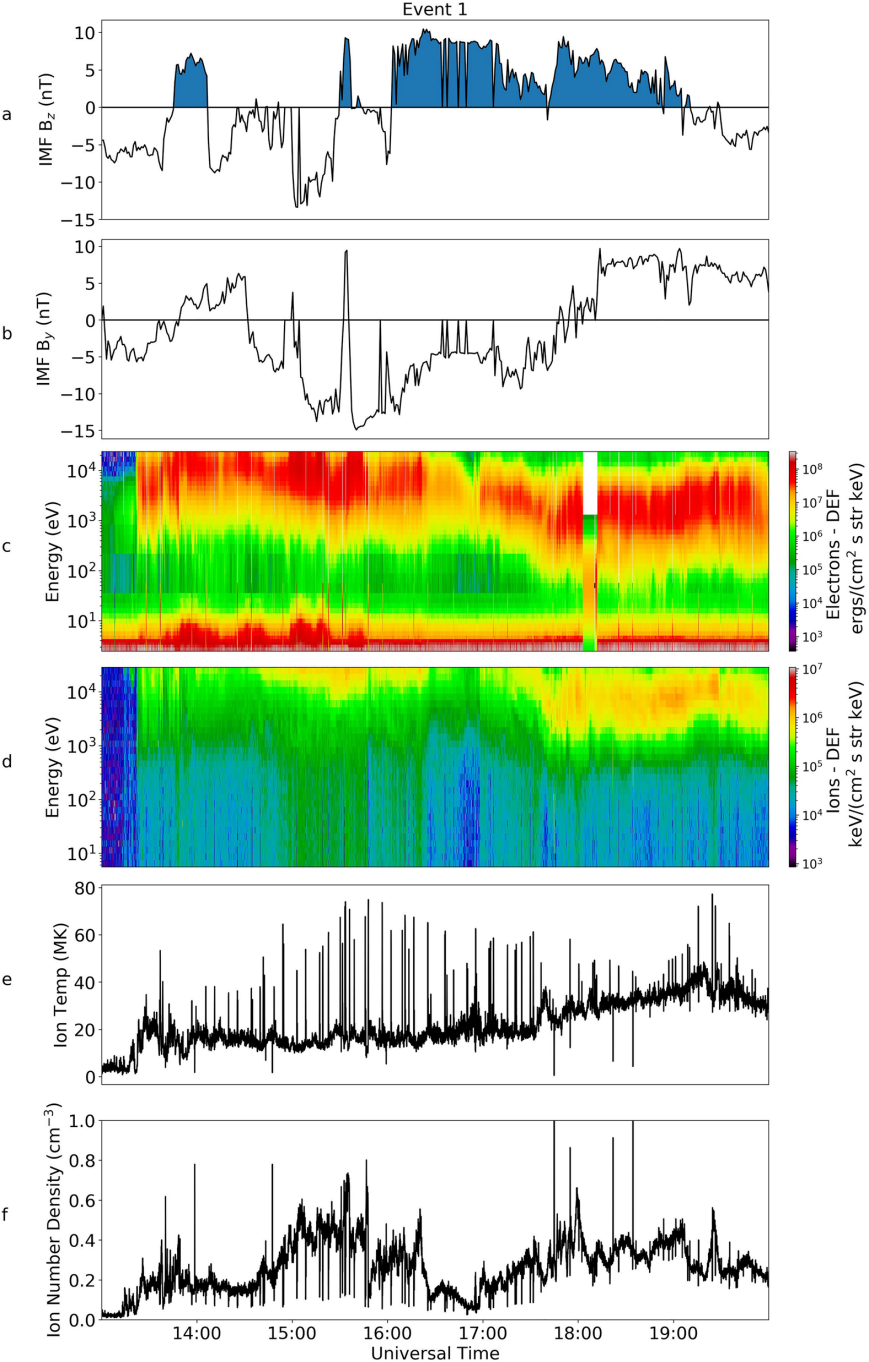
TC‐1 particle and magnetic field data for Event 1. Panel a and b show the B_*z*_ and B_*y*_ component of the Interplanetary Magnetic Field taken from the OMNI data set (first shown in Figures 3a and 3b). The electron differential energy flux spectrogram measured from Plasma Electron And Current Experiment instrument and the ion differential energy flux measured using Hot Ion Analyzer (HIA) is shown in panels c and d respectively. Panels e and f show the ion temperature and density respectively, also using HIA. These measurements are extracted from the on‐board moments.

The comparison made by Fear et al. (2014) with the plasma sheet was purely based on a statistical picture of the plasma sheet for northward IMF conditions (Walsh et al., 2013). However, TC‐1 was situated in the plasma sheet (at [−10, 0, 5] GSM) at 13:00 UT. In Figure 6, we present simultaneous observations from the TC‐1 HIA and PEACE instruments to investigate the inferences drawn in Fear et al. (2014). The figure shows the B_*z*_ and B_*y*_ component of the IMF as first seen in Figures 3a and 3b respectively. The spectrograms for the electron and ion energy distributions is shown in panel c and d; panels e and f detail the ion temperature and density.

At 17:00 UT TC‐1 observed a cooling in the plasma population (Figures 6c and 6d). The IMF had turned northward an hour prior to this (Figure 6a). If we compare the population observed by TC‐1 with that seen simultaneously by Cluster (Figures 3c and 3d), we see that the electron and ion energies observed in Cluster 1 are comparable to those observed in the plasma sheet by TC‐1 (averaging around 10^3^ and 10^4^ eV respectively). This similarity between the lobe and plasma sheet can also be seen in ion temperatures which peak at 40–60 MK. We note that the high energy tail of the ion population observed by TC‐1 before 17:30 UT (Figure 6d) was above the upper range of the instrumental operating mode, and hence not observed. Therefore, the ion temperatures in panel e prior to 17:30 UT are actually an underestimate of the true ion temperature, and so the apparent rise in temperature in Figure 6e is an artifact of this curtailment. In fact, as can be seen in the spectrograms, the plasma population was cooler after 17:30 UT than before. The densities recorded by TC‐1 show values which fluctuate about approximately 0.3 cm^−3^, though again the density before 17:00 UT is an underestimate due to the high energy truncation of the energy distribution. This is comparable to the magnitude of the densities measured by Cluster 1 shown in Figure 3f. This shows for the first time that the plasma characteristics observed by Cluster were similar to those observed in the plasma sheet at this time which substantiates the argument put forward in Fear et al. (2014).

### 3.2 Event 2

**Figure 7:**
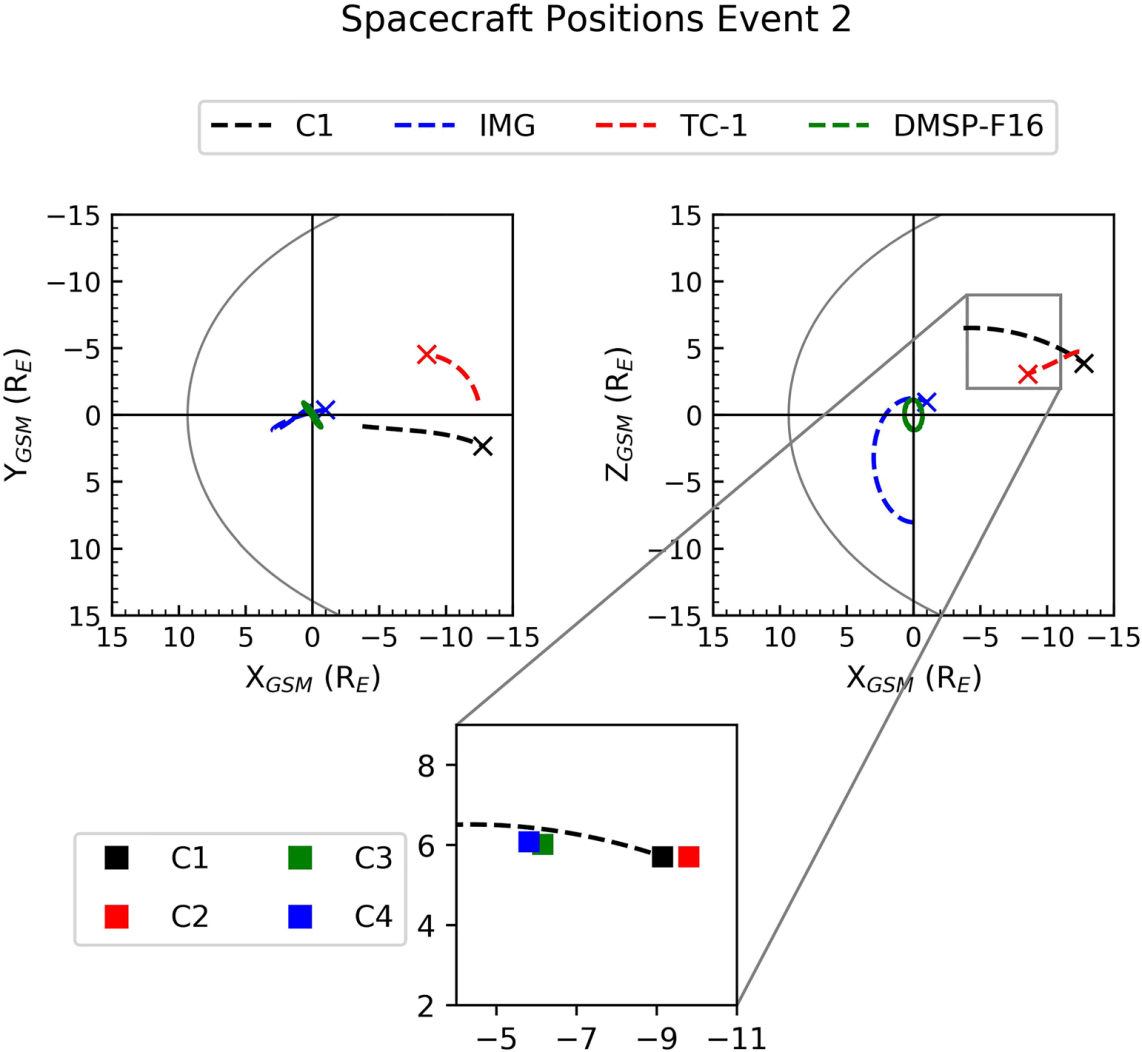
Orbit trajectories for Event 2 in the same format as Figure 2 with the addition of the position of all four Cluster spacecraft positions at 18:30 UT, represent by a square.

On September 30, 2005, the configuration of Cluster and TC‐1 was similar to Event 1, in that Cluster was positioned in the lobe region whilst TC‐1 was situated within the plasma sheet. However, whilst IMAGE was again in the Southern Hemisphere, Cluster 1 was now positioned in the Northern Hemisphere as can be seen in Figure 7. This figure also shows the separation of the Cluster quartet, which we will refer to later.

**Figure 8:**
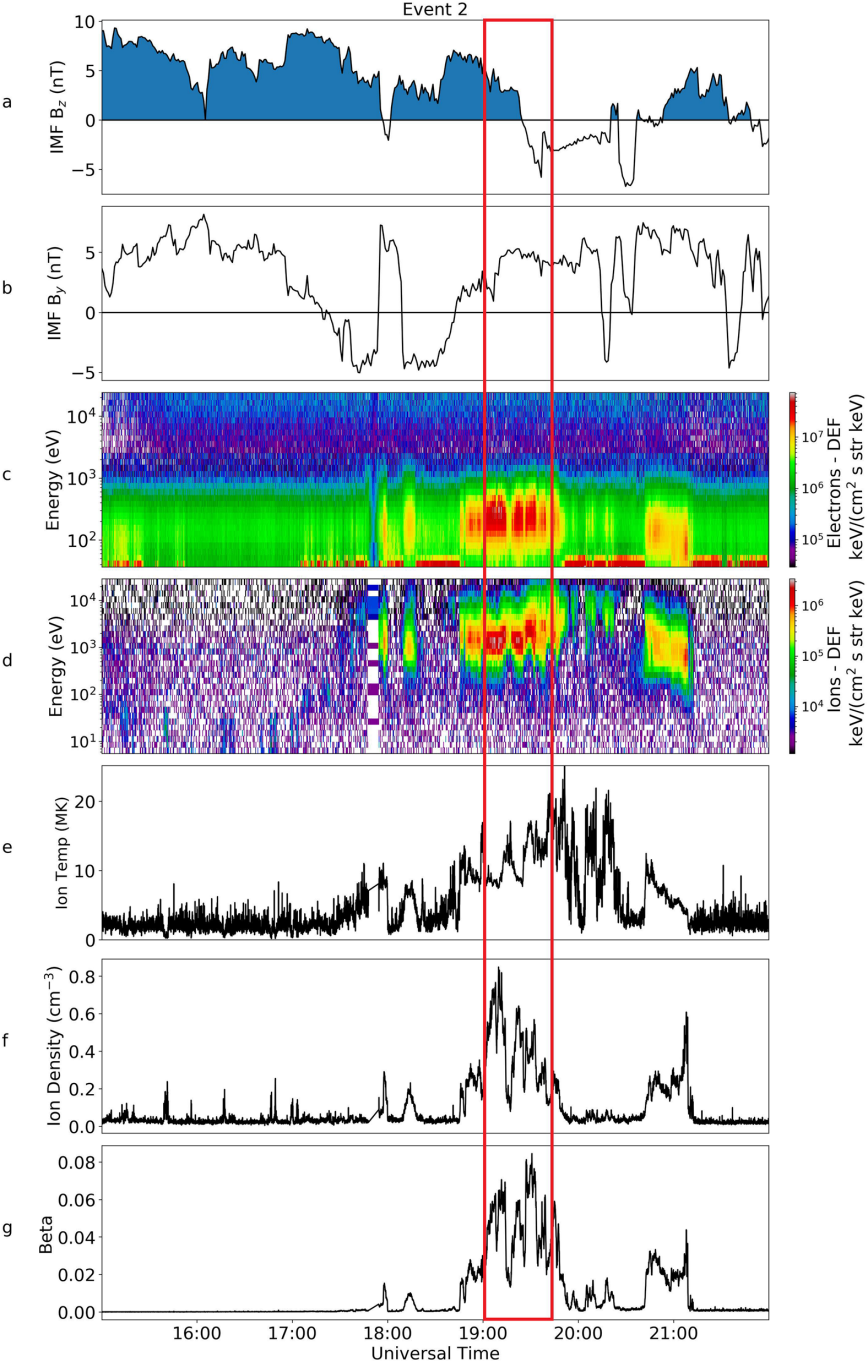
Magnetic and Particle data from OMNI and the Cluster 1 spacecraft for Event 2, in the same format as Figure 3.

During Event 2, Cluster 1 observed similar plasma characteristics to those in Event 1 (as shown in Figure 8). The IMF turned northward just before 15:00 UT (not shown) and continued to be northward until 19:30 UT. Over this period it can be seen that in the electron spectrogram (Figure 8c), there was a low energy background population with low differential energy flux (DEF) measurements. This was situated just above the spacecraft potential and hence corresponded to a natural plasma population rather than photoelectrons. This background population was almost entirely at energies below 1 keV.

At 18:00 UT Cluster 1 observed an increase in the electron DEF of the particles. At this time, an ion population appeared at higher energies (>1 keV). Prior to this, there was no detection of ions within the instrument's energy range. After 18:00 UT there were intermittent increases in the DEF in both the electron and ion data; there was a constant, more prolonged population between 18:45 UT and 20:00 UT which turned intermittent again until fading just after 21:00 UT. Shortly after the IMF turned southward, just before 20:00 UT, the ion temperature increases to a peak of 25 MK.

**Figure 9:**
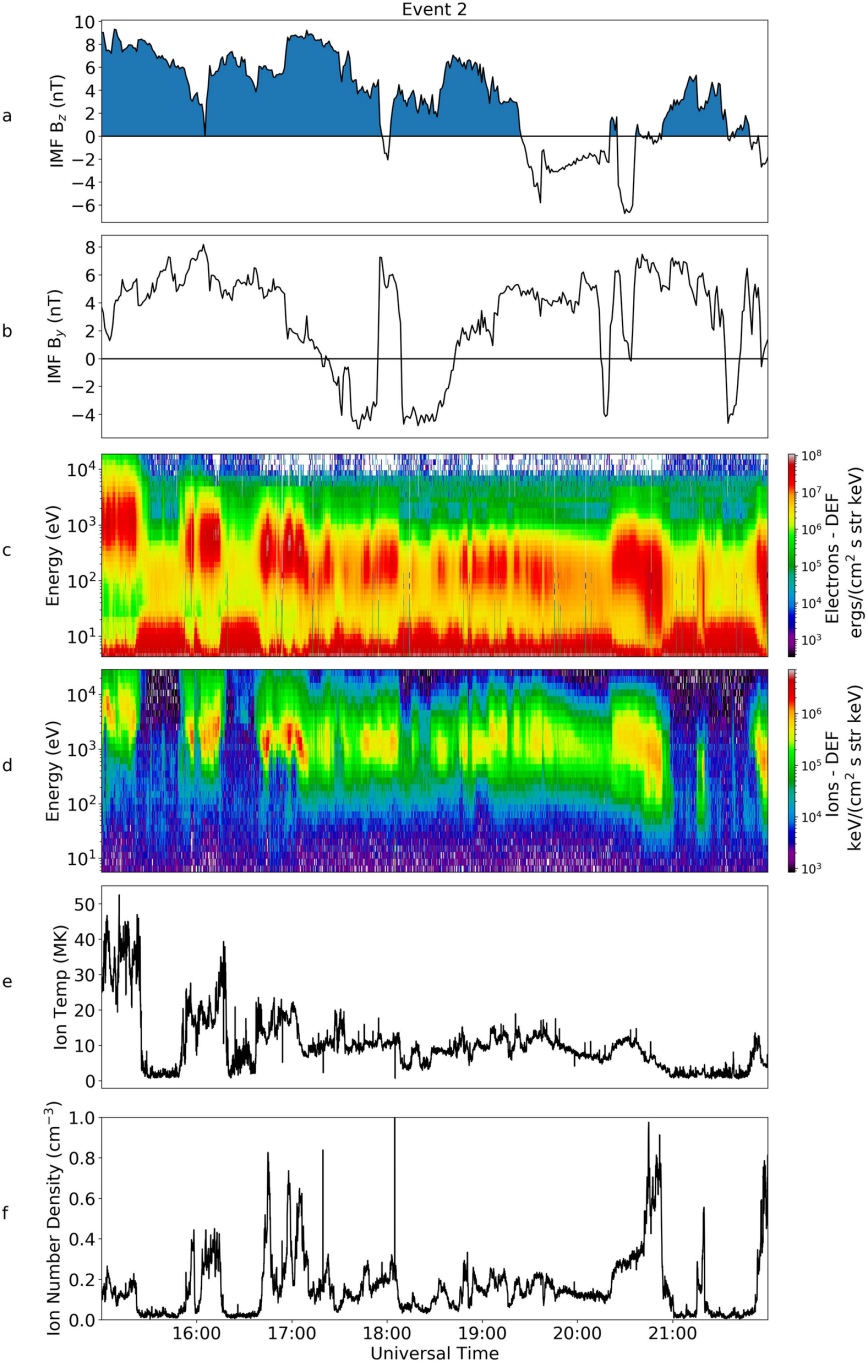
TC‐1 particle and magnetic field data for Event 2, in the same format as Figure 6.

TC‐1 observations of Event 2 are shown in Figure 9. As in the previous event, the plasma sheet was cooling at this time. The energy of the electron population at 15:00 UT was centered at about 10^3^ eV, but by 19:00 UT it had decreased by an order of magnitude to 10^2^ eV, comparable to the energies of electrons seen by Cluster 1. This decrease in energy was also seen in the ion spectrometer. The energies of the ions observed by Cluster 1 and TC‐1 were also comparable in magnitude. A corresponding decrease was observed in the plasma sheet temperature, from 40 MK at 15:00 to 10 MK after 17:00 UT. This compared well with the temperature observations by Cluster 1 (after 18:00 UT) which fluctuated between 20 and 10 MK (Figure 8e). The plasma sheet density remained fairly constant throughout the period of observation, rarely reaching over 0.5 cm^−3^.

**Figure 10:**
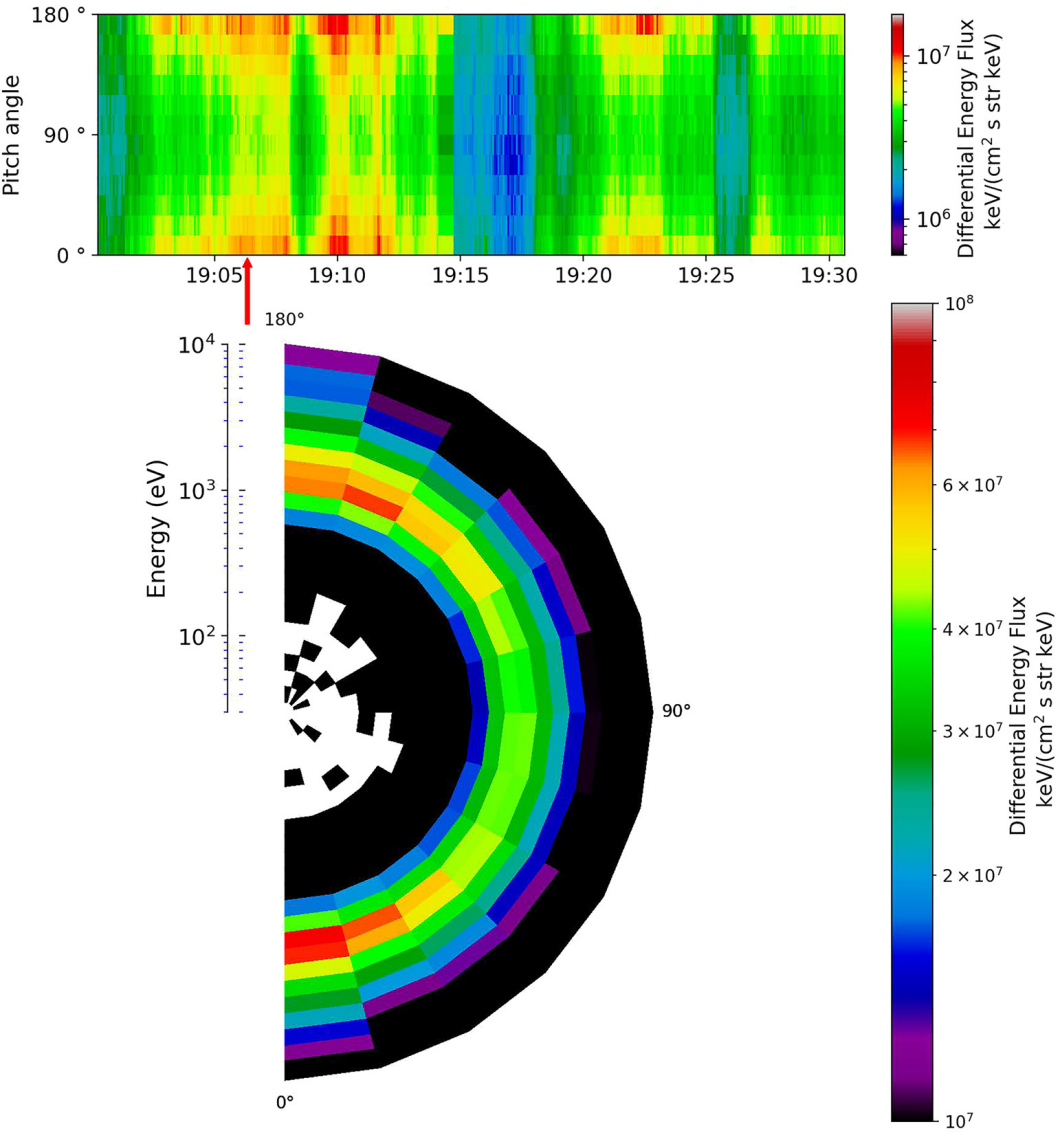
The electron pitch angle and energy distribution observed by Cluster 1 for Event 2, in the same format as Figure 4. The lower plot shows the energy with respect to pitch angle for an average of five time stamps centered about 19:06 UT (indicated by the red arrow).

Figure 10 (top) shows the PAD of the electrons from Cluster 1 over the period of 19:00–19:30 UT (region within the red box in Figure 8), as well as an average of five spins about 19:06 UT which can be seen at the bottom of this Figure, the specific time of which is indicated in the top panel by a red arrow. There was clear evidence of bidirectional electrons throughout the event (peaks in the DEF of electrons at 0° and 180°); an example of this can be seen clearly at 19:10 UT and later at ∼19:23 UT. We note a similarity to the bidirectional population observed for the majority of the interval for Event 1 (shown in Figure 4). Unlike in the first event, we observe no clear evidence for a double loss cone.

**Figure 11:**
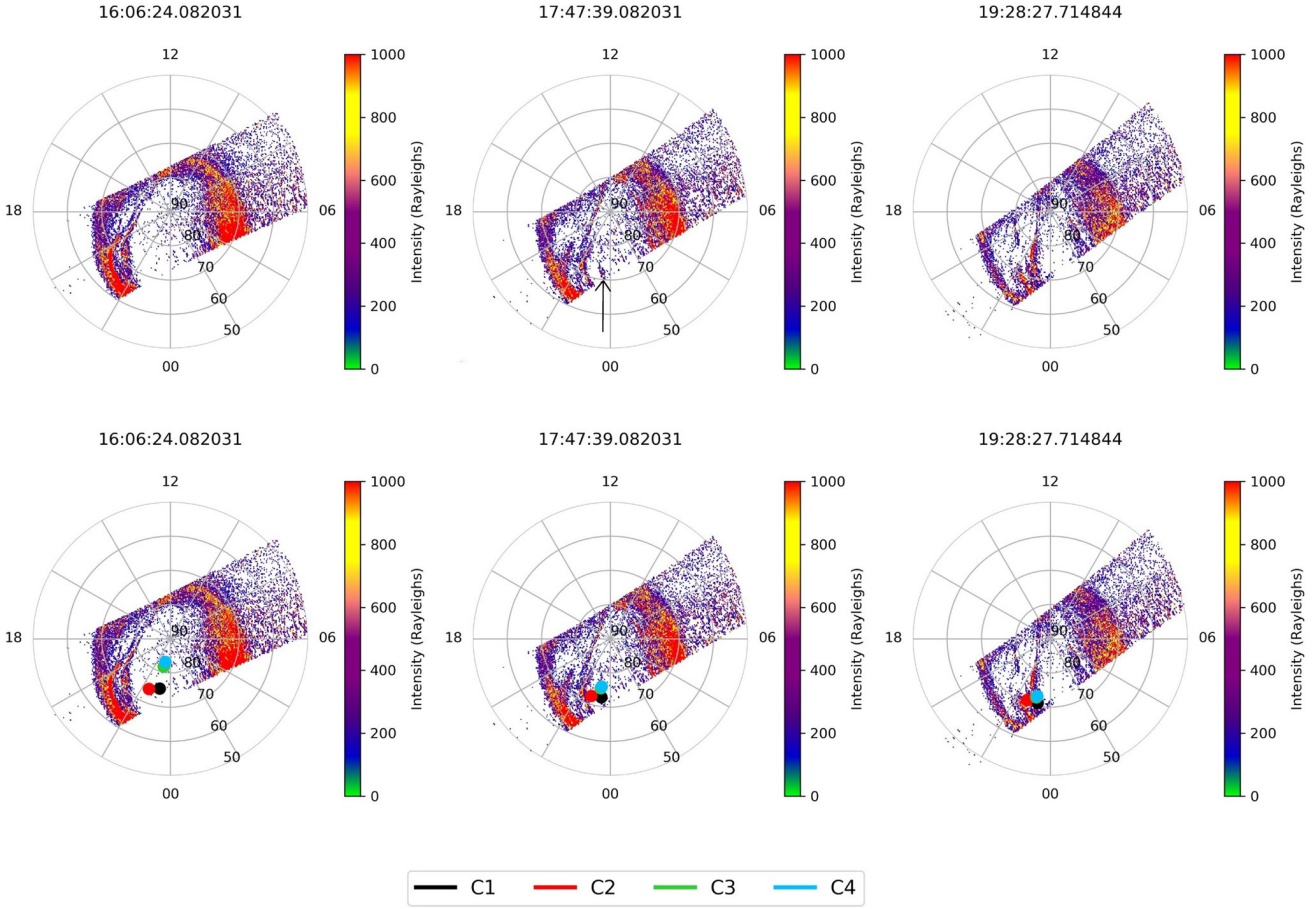
SSUSI (DMSP‐F16) Far Ultraviolet auroral observations from the Northern Hemisphere. The panels show the images taken from 16:06 UT to 19:28 UT for Event 2. The data are plotted in Altitude Adjusted Corrected Geomagnetic coordinates (magnetic latitude, MLT). The top three images are repeated in the bottom row but overplotted with the footprints of Cluster 1, 2, 3, and 4. This has been traced using the T96 model (Tsyganenko, 1996) to an altitude of 120 km and are represented by black, red, green, and blue circles respectively.

Global‐scale observations of the aurora are available for this interval from both IMAGE and the SSUSI instrument onboard DMSP‐F16. The DMSP spacecraft are in low Earth orbit, which allows us to observe both the Northern and Southern auroral regions, once every 100 min (with Northern and Southern observations from a given orbit being about 50 min apart). The SSUSI observations from the Northern Hemisphere (i.e., the same hemisphere as the Cluster observations) are shown in Figure 11; the top and bottom rows show the same three images, but the bottom row has been overplotted with the footprints of the four Cluster spacecraft. The images show the TPA has formed by 16:06 UT but at this time does not coincide with any of the Cluster spacecraft footprints (indicated by a corresponding colored circle depending on the spacecraft number). By 17:47 UT, we see that a larger structure spanned from the dusk‐midnight sector toward noon across the pole, just before it coincides with the footprint of the Cluster spacecraft. This is the time at which we begin to observe “hot” plasma in the lobe (18:00 UT) with Cluster 1, as seen in the ion and electron spectrograms in Figure 8. We then see a clear intersection between the footprint and the TPA at 19:28 UT in Figure 11, corresponding to the highest measured ion and electron DEF and energy values observed for this event (Figures 8c and 8d). The arc appeared to move dawnward but then reversed back duskward between 19:28 and 21:09 UT (not shown). The position of the TPA at 21:09 UT no longer coincides with Cluster 1.

**Figure 12:**
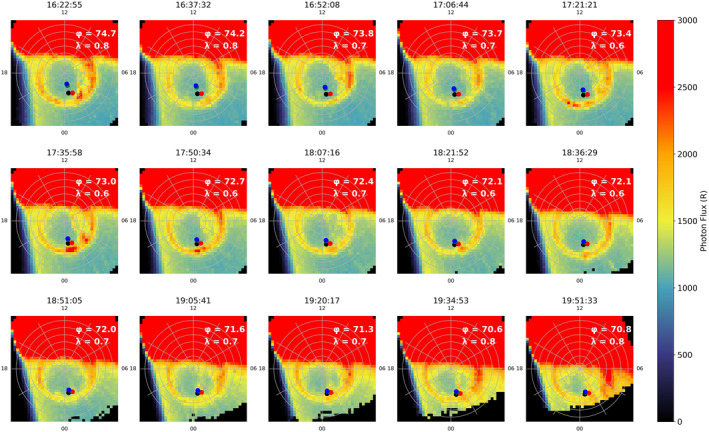
Imager for Magnetopause to Aurora Global Exploration Far Ultraviolet wide‐band imaging camera instrument data, observed the southern hemisphere for Event 2, in the same format as Figure 5. The southern hemisphere footprints of all four Cluster spacecraft are shown. Cluster 1, 2, 3, and 4 are represented by black, red, green, and blue circles respectively.

SSUSI also shows evidence for a TPA in the Southern Hemisphere, but this is seen more clearly in the observations provided by the IMAGE spacecraft. Figure 12 shows the photon flux of consecutive FUV (between 140‐160 nm) images from the IMAGE WIC. The first indication of an arc in the IMAGE data occurred at 16:22 UT in which an increase in photon flux at midnight in the auroral oval can be observed. Pre‐arc brightenings were also prominent in the mechanism proposed by Milan et al. (2005); they likewise occurred shortly before the appearance of TPAs. At 17:35 UT we see a polar cap arc forming on the dawnside which subsequently connects across to midnight. This structure then appeared to dissipate at 18:07 UT, as also seen by SSUSI in the South (not shown). A second oval brightening at 17:35 UT can be seen around ∼01 MLT. A second TPA forms at this position and spans into the polar cap, visible at 18:51 UT; at this time the footprint of the Cluster spacecraft also first appears to intersect the TPA. This arc then grew to higher latitudes but appeared to stay at this local time for the duration of the observation. After 19:51 UT, there are no IMAGE observations but we can confirm by comparison with SSUSI data that the arc was still present in both the Northern and Southern Hemispheres until at least 21:09 UT and 21:57 UT respectively (not shown). We observed a clear TPA spanning the entire polar cap with IMAGE in the South and SSUSI in the North, and note that the TPAs were on opposing sides of the noon‐midnight meridian, consistent with previous inter‐hemispheric observations of transpolar arcs (Carter et al., 2017; Craven et al., 1991; Reidy et al., 2018).

#### 3.2.1 Cluster 2, 3, and 4

So far, when investigating the plasma populations that are present in the lobes, we have focused on data from the Cluster 1 spacecraft. In Event 1, the difference between the data recorded from each of the four Cluster spacecraft was minimal due to the fact that all four spacecraft were within close proximity (<1R_E_). During Event 2, the separation between the spacecraft was larger (∼5 R_E_) in the X_GSM_ direction throughout Event 2, as shown in Figure 7. Cluster 1 and 2 were situated close to each other, as were Cluster 3 and 4, but the two pairs were separated by about 5 R_E_ in the X_GSM_ direction. By looking at the particle data for each Cluster spacecraft separately, we gain added spatial information for the first time. We utilize the separation in the Cluster spacecraft to observe the structure of the energetic plasma found in the lobe for Event 2.

**Figure 13:**
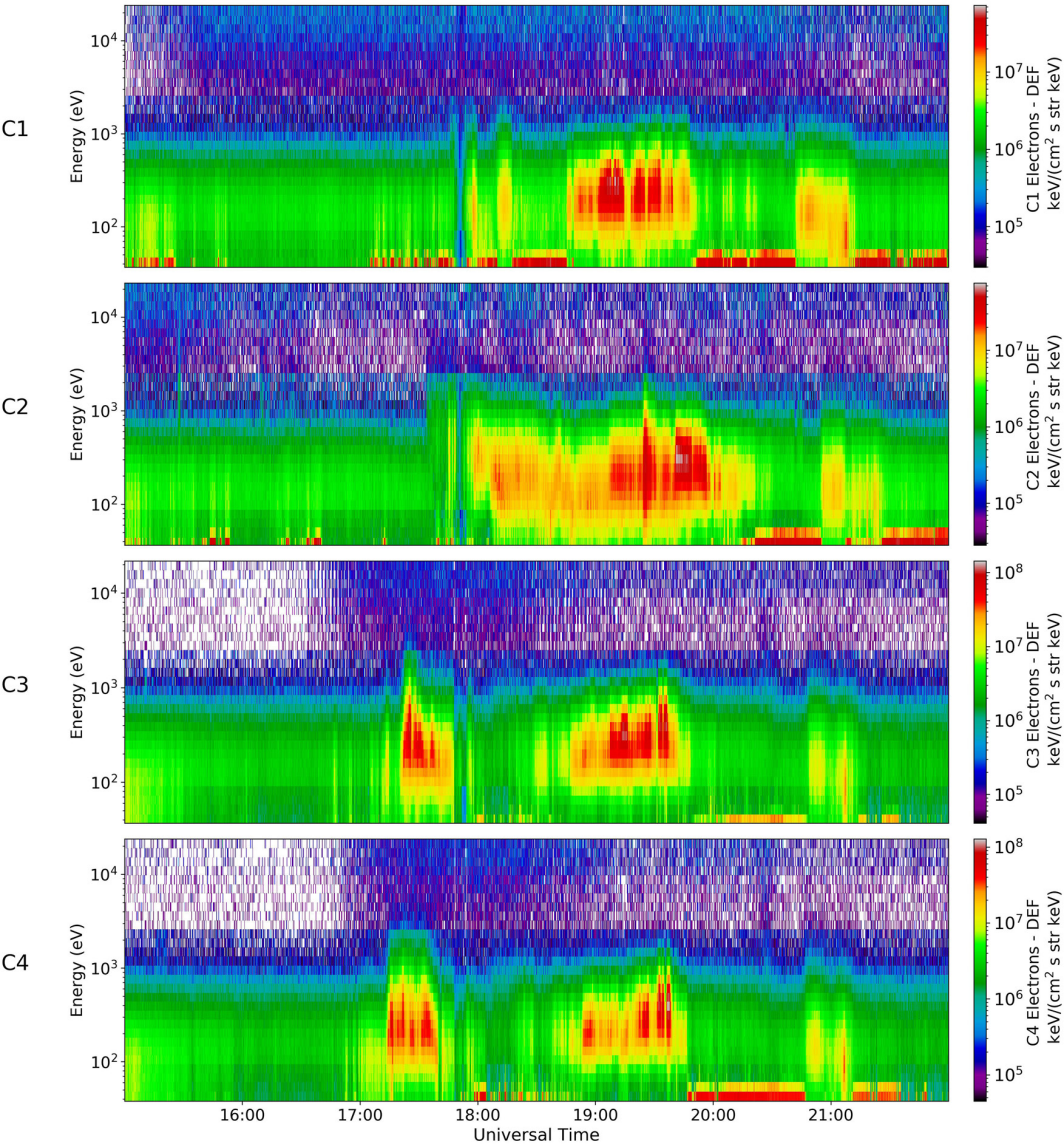
Electron particle data from Cluster 1, 2, 3, and 4 for Event 2. The energy spectrograms for each of the spacecraft, Cluster 1, Cluster 2, Cluster 3, Cluster 4 can be seen in panel 1, 2, 3, and 4 respectively. The C1 panel shows the same data as previously seen in Figure 8c.

Electron spectrograms for all four spacecraft can be seen in Figure 13. From these spectrograms, it appears that energetic plasma is first observed by Cluster 4 (just after 17:00 UT), and soon followed by Cluster 3. The population had relatively high differential energy flux and the maximum energy measured for this population was ∼10^3^ eV. This population was observed for ∼30 min. A similar population was then observed by Cluster 2, but we note that the onset of this population occurred just after the plasma population disappeared in Cluster 3 and 4, at around 18:00 UT. This population had a broadly constant energy for the duration of the interval, peaking at 10^3^ eV. The plasma that was observed by Cluster 2 was present for just over two hours, the longest continual observation out of all four spacecraft, and tailed off just after 20:00 UT. The population was observed last by Cluster 1, predominantly between 18:45 UT and 19:45 UT. This plasma had comparable energies and DEF values to that observed by all other spacecraft, but was somewhat short lived with respect to Cluster 2 observations.

We can interpret our multi‐spacecraft in situ observations with the aid of the auroral images that were discussed above (Figures 11 and 12). We first consider the auroral observations from IMAGE (Figure 12), given their higher cadence (though we note that these are observations from the opposite hemisphere from Cluster). The first arc discussed above formed on the dawnside at ∼03 MLT at 16:22 UT. This continued to form into a TPA which spanned from midnight to noon and appeared to stay on the dawnside of the polar cap. This arc does not intersect any of the Cluster spacecraft and hence we do not observe any corresponding particle distributions in Figure 13 before 17:00 UT. At 17:35 UT we observe an auroral brightening at ∼01 MLT (as discussed above). The configuration of the spacecraft at this time led to a notable separation in their footprints, with Cluster 2 being the most dawnward (nearing 02 MLT). Cluster 1 can be seen to have the same latitude as Cluster 2 but was closer to midnight MLT than Cluster 2. Cluster 3 and 4 were relatively close to each other and cannot be distinguished clearly in this plot. They are located at a higher latitude and have the same local time as Cluster 1. None of the spacecraft intersect with the oval as the spacecraft are located at latitudes higher than 70° MLAT which appears to mark the poleward boundary of the oval at this time. By 18:21 UT a second TPA formed from the brightening at 01 MLT and it began to protrude into the polar cap. Cluster 2 appears to be located at the same local time and latitude as the TPA at 18:51 UT. This is the first intersection of the TPA by any of the Cluster spacecraft that was visible in IMAGE data. By 19:05 UT, Cluster 1, 3, and 4 map directly on top of the auroral brightening. From the IMAGE data it is not clear where the initial briefly observed population observed in Cluster 3 and 4, between 17:00 UT and 18:00 UT, originates.

We examine SSUSI data, which despite the lower cadence, offers high spatial resolution and is in the same hemisphere as Cluster. The lower panels in Figure 11 show the position of the Cluster spacecraft at three consecutive time intervals. As in the Southern Hemisphere, at 16:06 UT there is no intersection of the spacecraft and hence no plasma observations. By 17:47 UT, we see the positions of all the spacecraft have moved equatorward toward the oval and the TPA discussed above is on the duskside (opposite to that seen in IMAGE, as predicted to occur in the mechanism by Milan et al., 2005). Here the SSUSI observations are consistent with the IMAGE data (Figure 12) in that the Cluster 2 spacecraft intersects the arc first. However, if the footprints are removed (top row), it can be seen that there is a smaller, secondary arc, which lies directly under the Cluster 3 and 4 spacecraft footprint at 23 MLT (indicated by an arrow in Figure 11), not captured by IMAGE. This can explain the observations of a short plasma population which ends just before 18:00 UT, and is only observed by Cluster 3 and 4 (Figure 13). The Cluster 1 footprint appears to be at the same local time as Cluster 3 and 4, hence initially it might be questioned why there was not a more prominent plasma population observed in the particle data. From closer inspection, it can be seen that Cluster 1 maps more equatorward than Cluster 3 and 4 hence does not directly pass through the smaller, secondary arc (which has an east‐west component to its alignment). By 19:28 UT the secondary arc appears to have either merged with the larger TPA, seen at 23 MLT, or disappeared. At this time, all four spacecraft coincide with the TPA seen by SSUSI, which corresponds to the most energetic plasma populations measured by all the Cluster spacecraft in Figure 13. The fact we observe corresponding intersection times between the TPA and footprints, with the Cluster particle data, further supports the link between plasma observations which are observed at high latitudes in the lobe and the formation of global transpolar arcs. These observations are also consistent with previous plasma characteristics seen in Event 1. These observations confirm our interpretation of this event which show there is a direct link between the TPA formation in the Northern and Southern Hemispheres and the uncharacteristically hot and dense plasma observed in the lobe.

### 3.3 Event 3

**Figure 14:**
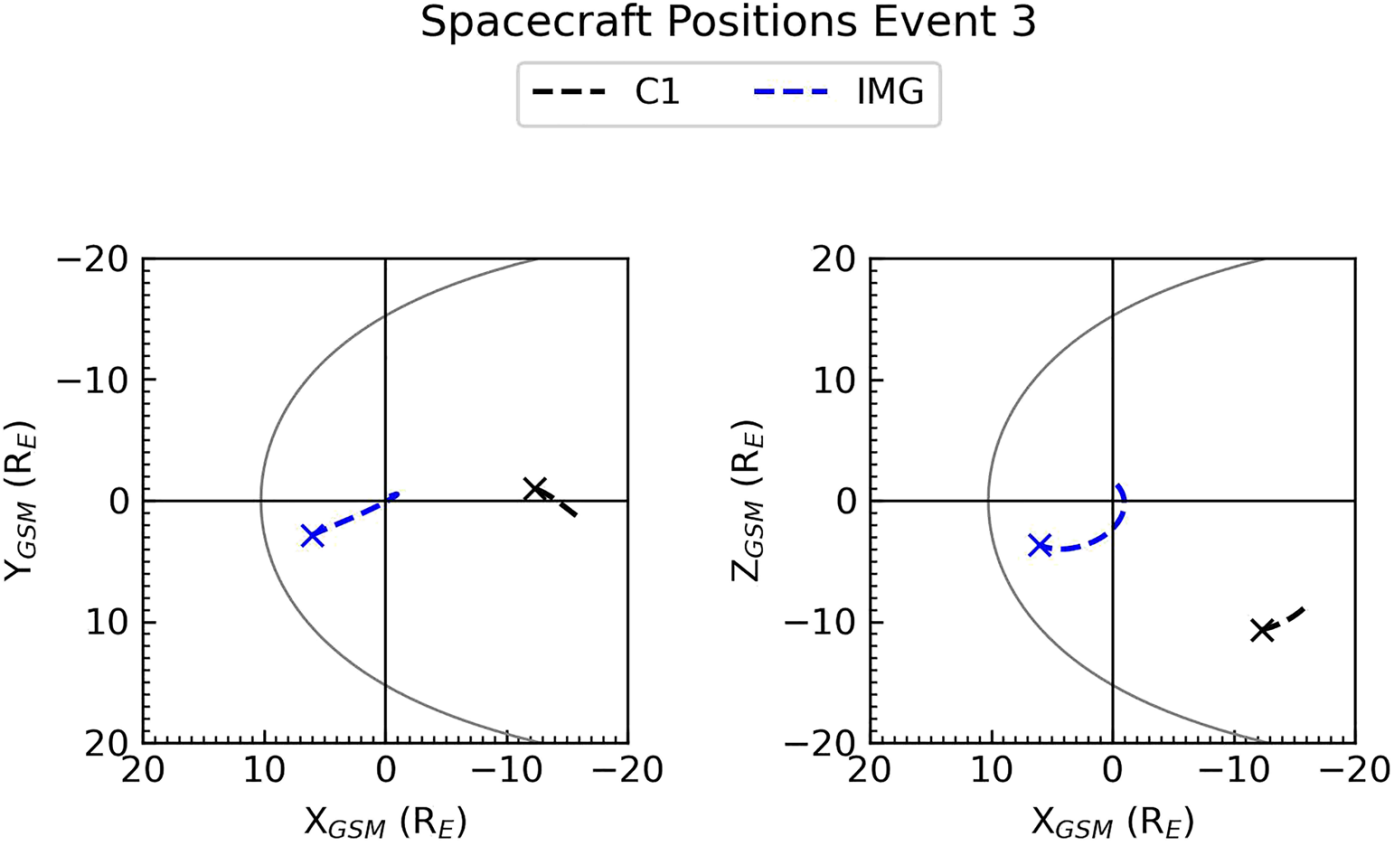
Orbit trajectories of the spacecraft used to investigate Event 3. The trajectory of Cluster 1 and Imager for Magnetopause to Aurora Global Exploration can be seen in black and blue respectively. The asterisk marks the end of the orbit at 09:00 UT, with the same format as Figure 2.

The final event we will study comes from the September 11, 2003 between 04:00 and 09:00 UT, when uncharacteristically “hot” plasma was observed in the lobes, similar to Event 1 and 2. Here we observe that Cluster is again in the lobe regions but is positioned further downtail than the previous two events, as shown in black in Figure 14. No simultaneous plasma sheet observations were available for this event as it occurred before the launch of Double Star, however, a comparison with the preceding plasma sheet crossing by Cluster can be seen in Figure S3 in the supplementary information. DMSP‐F16 was also not in orbit at the time of this event but IMAGE provided Southern Hemisphere observations of the aurora; the trajectory of IMAGE can be seen in blue in Figure 14.

**Figure 15:**
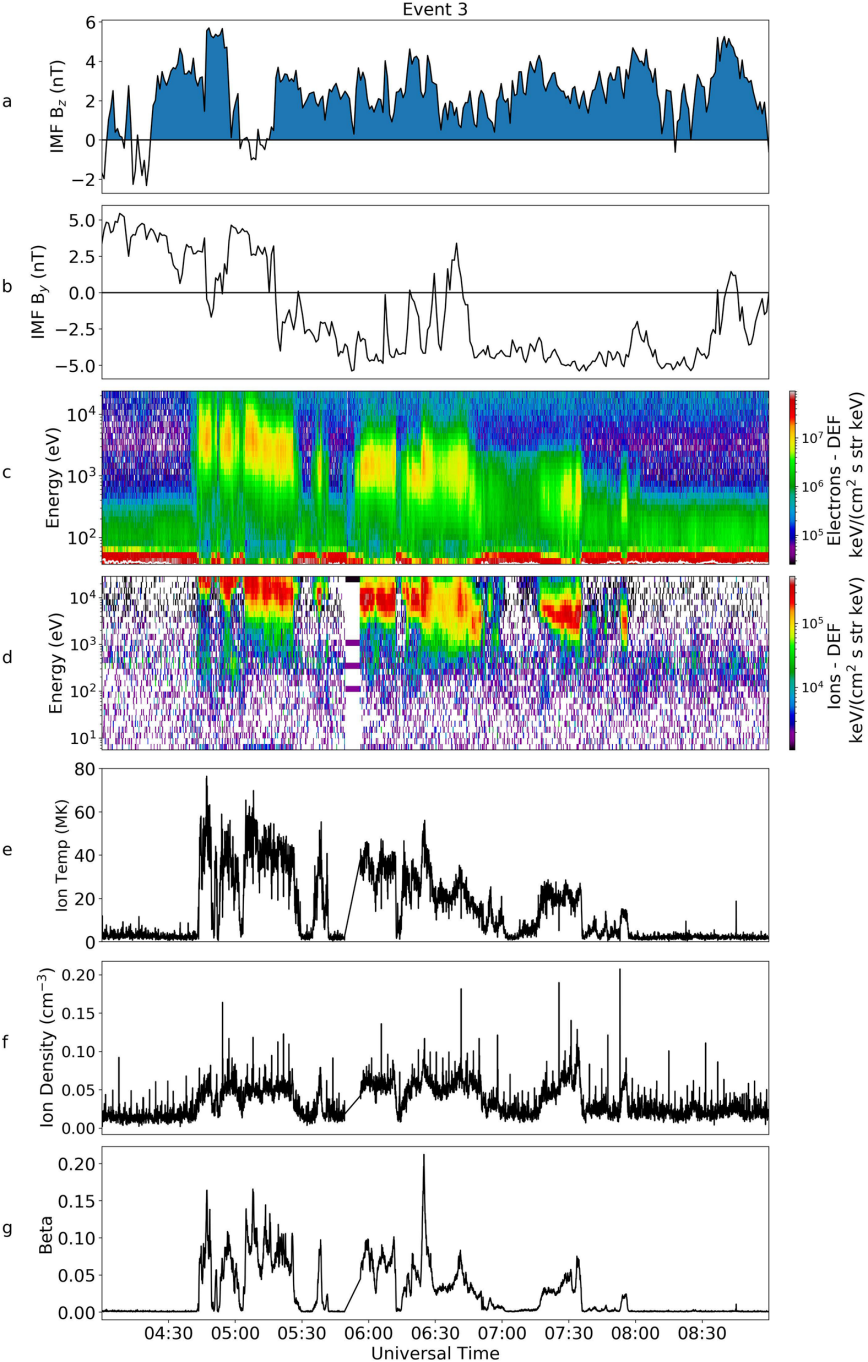
Magnetic and Particle data from OMNI and the Cluster 1 spacecraft for Event 3, in the same format as Figure 3.

Figure 15 presents the data from Cluster 1. We can see from Figure 15a that the IMF turned northward just prior to 4:30 UT. There was a brief southward turning at 5:10 UT, for approximately 10 min, but the IMF stayed continuously northward after this time until 9:00 UT. We note that during this interval there was a sharp change in the B_*y*_ component from positive to negative at 05:30 UT. The energy of the electrons was initially of the order 10^4^ eV but steadily declined to below 10^3^ eV. Similar energies were observed in the ion spectrogram but they were an order of magnitude higher, as observed for all the previous events (Figure 15d). This decline is clearly seen in the temperature of the ions (Figure 15e). There were small fluctuations of density over this time but no significant overall increase or decrease was observed (Figure 15f).

**Figure 16:**
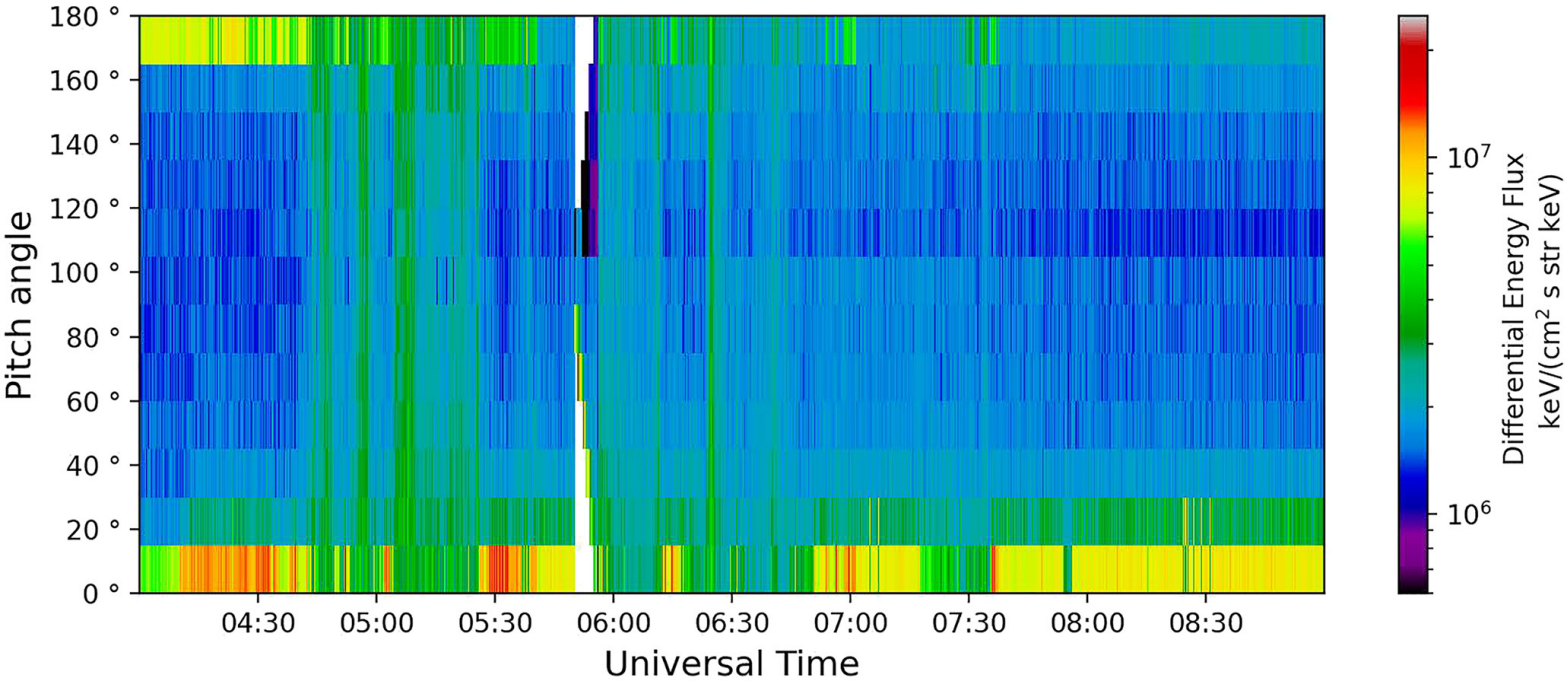
The electron pitch angle distribution, measured by Plasma Electron And Current Experiment on‐board Cluster 1 for Event 3.

The electron PAD, seen in Figure 16, shows bi‐directional properties similar to that seen in Events 1 and 2. There were periods which showed a more isotropic electron distribution, visible at 04:45 UT, just before 05:00 UT, just after 05:00 UT and again at 06:25 UT, but there was no evidence for a double loss cone at these times.

**Figure 17:**
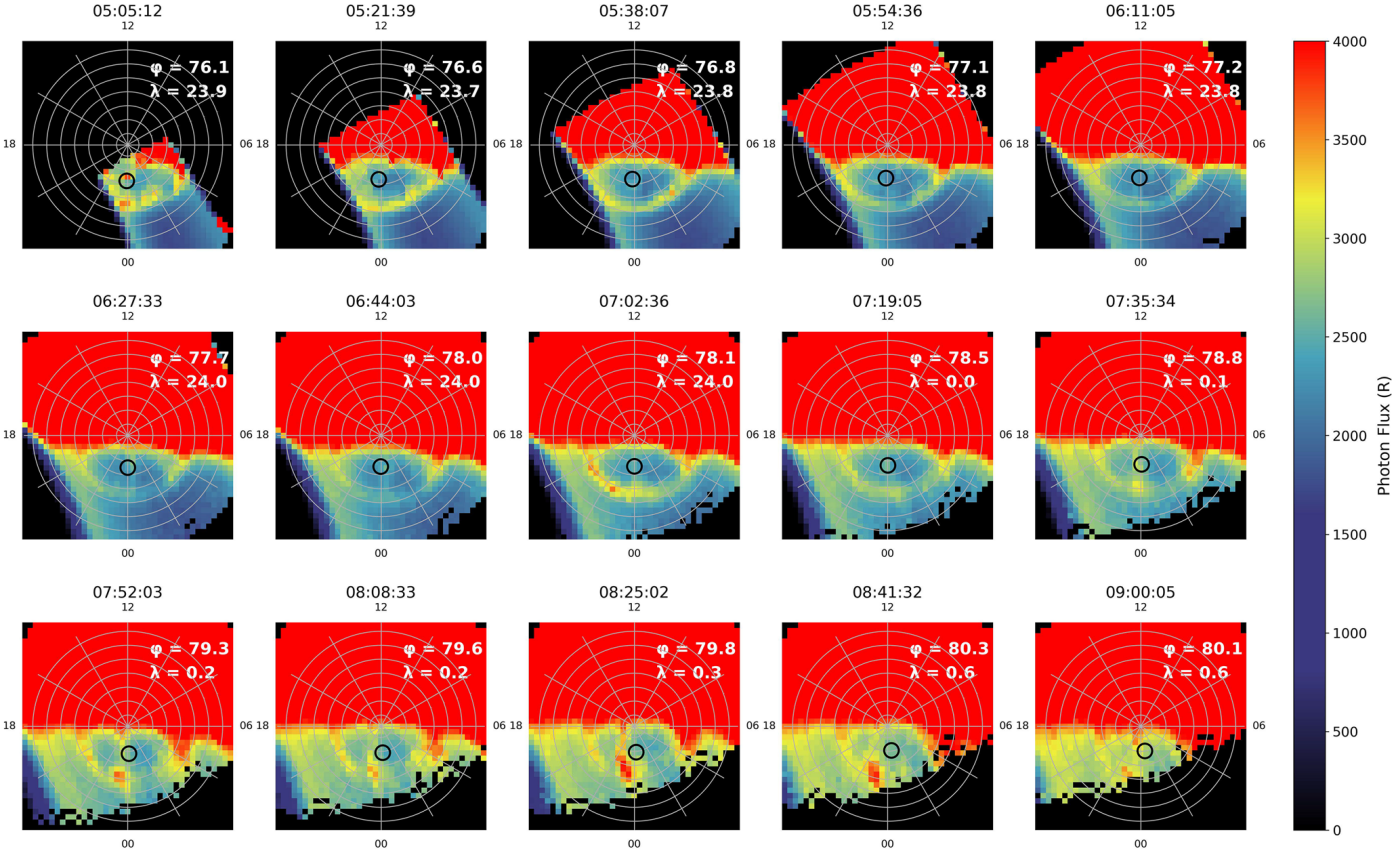
Imager for Magnetopause to Aurora Global Exploration (IMAGE) Far Ultraviolet wide‐band imaging camera observations of the Southern Hemisphere during Event 3. This figure has the same format as Figure 5. The panels shown each correspond to the field of view that IMAGE had of the pole, each separated by ∼15 min interval between 05:05 UT, when the southern pole just came into the view of IMAGE and 09:00 UT when the polar cap was no longer in view. The black hollow circle represents the Cluster 1 mapped footprint at 120 km altitude in Altitude Adjusted Corrected Geomagnetic coordinates.

The Southern Hemisphere aurora was observed by IMAGE at this time, but the quality of the images was poorer (due to dayglow contamination). We observe in Figure 17 that there is evidence for a TPA at 05:05 UT underneath the Cluster 1 footprint (shown as a hollow circle to allow the corresponding auroral emission to be seen). The observations become clearer as the IMAGE spacecraft moves to higher altitude in its orbit, meaning the field of view over the polar region is increased. The arc forms in the Southern Hemisphere, and can be seen aligned along the noon‐midnight meridian. The TPA increases in brightness from 05:05 UT until 07:52 UT. Throughout this period the Cluster 1 footprint lies directly on top of the arc and we see a coincident high‐energy plasma population in the PEACE and HIA spectrograms measured by the Cluster 1 spacecraft (Figures 15c and 15d). The TPA then appears to move duskward and hence is no longer positioned under the Cluster 1 footprint from about 08:08 UT. This coincides with the disappearance of the hot plasma population observed by Cluster 1 at this time (Figure 15), though IMAGE observed the TPA (duskward of Cluster 1) until just after 08:51 UT. There is no IMAGE data after this time due to the equatorward motion of the spacecraft orbit, hence reducing the field of view of the pole. A short southward turning of the IMF occurred at 9:15 UT and then the IMF was persistently southward after 11:00 UT (not shown), at which point we would expect the TPA to fade.

## 4 Discussion

### 4.1 Observational Summary

We have presented three events when uncharacteristically “hot” and dense plasma has been observed in the lobes of the magnetosphere. The observations all showed a turning of the IMF to northward, shortly followed by a presence of “hot” plasma. The three events showed different energy characteristics. For Event 1, we observed the most energetic plasma (10^3^ eV for electrons and 10^4^ eV for ions). In Event 2, we observed energies that were an order of magnitude lower for both ions and electrons, of which the plasma energies also remained constant with time. In the last case study, Event 3, the energy of the plasma in the lobe decreased over time from nearly 10^4^ eV to under 10^3^ eV for electrons, and from above 10^4^ eV to just over 10^3^ eV for ions.

In Events 1 and 2 we compared the characteristics of the “hot” plasma observed by Cluster, with those found in the plasma sheet by TC‐1. For both of these events, the energy of plasma embedded in the lobe was comparable to that of the relatively cool plasma sheet. For Event 3, in which we had no comparable plasma sheet values, we observed similar orders of magnitude to the other events indicating this too was likely consistent with the plasma sheet.

We observed evidence of TPAs or polar cap arcs in all three case studies. For Event 2 we observed clear conjugate TPAs which spanned the entire polar cap (observed by IMAGE in the southern hemisphere and SSUSI in the north). For Events 1 and 3 we had clear observations of an arc in the same hemisphere as that of the Cluster observations. For each event, there were multiple intersections between the TPA and the Cluster 1 footprint, the times of which all corresponded to the presence of plasma observed by Cluster 1 in the lobe.

### 4.2 Analysis

We have presented observations of three events which show a link between transpolar arcs and “hot” plasma embedded in the magnetotail lobes. We have shown for the first time that hot plasma exhibits the same characteristics as simultaneous observations of the plasma sheet during these events, as well as investigating the spatial characteristics of the arcs using the Cluster tetrahedron for one event, substantially advancing the argument of Fear et al. (2014).

Considering the hotter population alone (i.e., excluding the possibility of a cold ion population which can be observed in the lobe below the energy range of the HIA instrument), these plasma populations are also denser than the typically sparse plasma which is observed at such energies in the lobe. We observed differences in the peak energies that were measured for each event, with the first event being the most energetic. Event 2 saw cooler temperatures over the entire period compared to those measured for Event 1. The overall energies observed by Cluster and TC‐1 for Event 2 were an order of magnitude lower than those observed in Event 1, but we note that in both cases the energy and temperature of the plasma populations observed by Cluster corresponded well with the equivalent parameters in the plasma sheet (observed by TC‐1), indicating that the difference between these two days was a global response to a different history of geomagnetic driving conditions.

Both Events 1 and 2 saw a gradual cooling of the plasma sheet which occurred after the IMF turned northward; this can be seen in Figures 6 c, 6d, 9c, and 9d at 17:00 UT and 19:00 UT respectively. The cooling of the plasma sheet is superficially suggestive of Cold Dense Plasma Sheet (CDPS) conditions (Taylor et al., 2008); however, the plasma sheet temperatures seen in Event 1 did not reach temperatures of less than 1 keV, nor densities above 1 cm^−3^ which are typically used to define CDPS (Fuselier et al., 2015; Øieroset et al., 2005). The plasma sheet in Event 2 had an average temperature of 10 MK at a time when we observed conjugate plasma with Cluster in the lobe. This is just within the temperature threshold for CDPS conditions, but the plasma density did not exceed 1 cm^−3^ at any stage so we conclude it is also not consistent with typical CDPS observations. We therefore do not interpret the cooling of the plasma sheet observed by Double Star in Events 1 and 2 as the formation of CDPS, but instead we view it simply as the transition from the hotter state that is typical of southward IMF conditions to a cooler state that is typical of northward IMF (Fujimoto et al., 2005; Petrukovich et al., 2003; Walsh et al., 2013)—presumably as a result of the reduced convection (Burch et al., 1985; Heelis et al., 1986; Reiff & Burch, 1985) and geomagnetic activity that arises under northward IMF conditions (Hoffman et al., 1988). We note in passing the similarity of both the ion and electron energies in Event 2 (and the plasma sheet observed simultaneously by TC‐1) to those in the case study reported by Shi et al. (2013).

For Event 3, we do not have contemporaneous observations of the plasma sheet however, the peak energies observed by Cluster 1 are similar to those observed in Event 1 as we saw peaks in DEF of electrons and ions at energies of 10^3^ and 10^4^ eV respectively. The lowest temperatures observed in this event, toward the end of the interval of interest, were more comparable with Event 2. This indicates that the energies were consistent with previously observed plasma sheet measurements and fall within the expected range of typical plasma sheet values for northward IMF despite not having a direct comparison.

**Figure 18:**
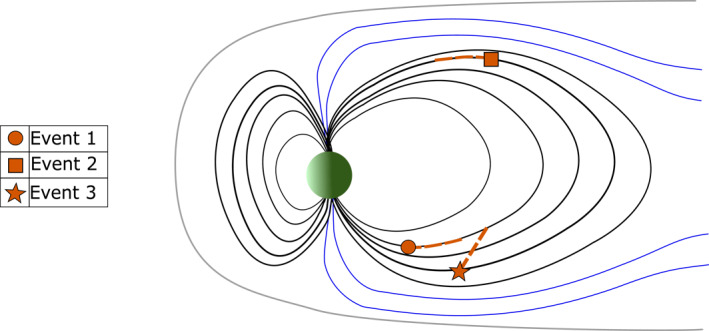
Schematic of the closed field lines which represent the “wedge” that forms as a result of tail reconnection due to northward Interplanetary Magnetic Field (IMF) conditions with a non‐zero B_*y*_ component as described by Milan et al. (2005). The trajectory of Cluster during each event is represented by a red dashed line. The marker denotes the end of the trajectory of the spacecraft. Blue field lines represent the field lines which are open and have not yet undergone tail reconnection (as shown in Figure 1 [left]). The black field lines represent closed field lines and on the nightside show the expected “wedge” of closed flux which forms at a discrete local time in the tail, as a result of a tail reconnection during northward IMF (Milan et al., 2005).

We now discuss the consistency of the plasma energies in the three events with the Milan et al. (2005) mechanism with reference to the schematic shown in Figure 18, which is based on Figure 1 (left) but includes representative spacecraft trajectories for the three events. To do this we look at the latitudinal and local time variation of the spacecraft footprint, which is stated in the corner of each panel in Figures 5, 12 and 17. For Events 1 and 2, the energy of the plasma population remained relatively constant throughout each period of interest. In Event 1, between 18:12 and 18:54 UT (corresponding approximately to the red box in Figure 3), the magnetic latitude and MLT of the footprint remains steady at 84 ± 0.3° MLAT and 13.5 ± 0.2 MLT. For Event 2, the MLT and latitude of the Cluster 1 footprint also remained steady during the period in which Cluster observed the energetic plasma population (18:00UT to 21:00UT Figure 8), at 71.5 ± 1° MLAT and 0.7 ± 0.1 MLT. Therefore, in both cases the limited variation in the footprint indicated that the motion of the spacecraft perpendicular to the magnetic field is minimal, and hence the spacecraft are essentially sampling the same field lines throughout the two intervals (and the energy of the plasma on those two field lines remains constant).

Event 3 is different, as the plasma distribution observed by Cluster declined in energy (and therefore temperature) throughout the interval (Figure 15). We interpret this cooling as a spatial effect and attribute it to spacecraft motion whereby the spacecraft moves onto field lines that contain cooler plasma. This is evidenced by Figure 17, which shows that the footprint of Cluster 1 moved ∼4° poleward during the period in which the hot plasma was observed by Cluster (05:00 to ∼08:00 UT), whilst the local time of the spacecraft stayed steady at 0 ± 0.3 MLT. This indicates that there is a significant component of motion of the spacecraft perpendicular to the magnetic field, suggesting that the spacecraft crosses closed field lines in the lobe (moving from field lines which map to low latitudes, to field lines which map to high latitudes within the wedge of closed flux [Milan et al., 2005]). We represent this scenario with the trajectory that ends in a star in Figure 18. The poleward motion, coupled with a decrease in energy of the plasma population over the duration of the event, is consistent with Milan et al. (2005) whereby the movement onto higher latitude field lines which are less contracted, results in the plasma populations being cooler.

This result conflicts with the mechanism presented by Shi et al. (2013). In that mechanism, the convecting open lobe field lines would be stretched as the IMF drags them around to the nightside. As a result, we would expect observations of plasma that map further toward the nightside of the ionosphere, where the IMF has significantly stretched them, to be cooler (Figure 1). Instead, we observe a cooling plasma population as the spacecraft moves onto higher latitude field lines away from the plasma sheet. With effectively single spacecraft observations of the lobe structure (since the Cluster tetrahedron was small for this event), we cannot completely rule out that the decrease in energy of the population observed by Cluster in Event 3 was a temporal effect. But given the lack of cooling observed in the TPA plasma for Events 1 and 2 (over a similar time frame and for similar IMF conditions) and the greater extent and longer duration of the cooling observed in the TPA plasma for Event 3 (compared with the cooling seen in the plasma sheet for Events 1 and 2), we think it is reasonable to infer that the cooling observed for the TPA plasma in Event 3 is more likely to be due to the spatial effect of the spacecraft motion.

The three events exhibit similar characteristics in their electron PADs, although perpendicular electron pitch angle distributions only dominate in Event 1 (Figure 4). This is indicative of a double loss cone which arises on closed field lines (Fear et al., 2014). These conditions are typically observed, even during northward IMF, in the inner plasma sheet, where the plasma beta parameter is typically greater than one (Walsh et al., 2013). However, even for Event 1, the plasma distribution was bi‐directional for the majority of the time. This is consistent with plasma populations observed at the outer plasma sheet which have not yet isotropized.

For Event 2, we observed no evidence for a double loss cone but the PAD contained both bi‐directional electrons and periods when the distribution was more isotropic, suggesting a possible transition between bi‐directionality and a double loss cone as reported by Walsh et al. (2011), Walsh et al. (2013), and Fear et al. (2014). We suggest that the difference seen in the PAD between Event 1 and 2 can be explained by the difference in time since tail reconnection occurred and the relative location of the spacecraft. The plasma in Event 2 is interpreted as being on newer closed lobe field lines, meaning that the plasma distribution has not yet had time to isotropize at the location of the spacecraft (Walsh et al., 2013). This is comparable to the process of forming the plasma sheet, in which bidirectional plasma is observed in the plasma sheet boundary layer (corresponding to the most recently closed field lines), and then transitions to an isotropized population which rapidly develops a double loss cone (e.g., Walsh et al., 2013).

The PAD for Event 3 was mostly bidirectional. There were times when we observed an isotropic distribution but they were brief. This is consistent with the statistical pattern seen by Walsh et al. (2013), when transitioning from the outer plasma sheet boundary region to the inner plasma sheet regions. Here we conclude that we were, relatively speaking, further out in the extended plasma sheet structure than the previous two events.

The complexities observed in these three cases illustrate that pitch angle distributions alone can be insufficient to identify the topology of field lines containing such populations. A bidirectional population, as observed through most of the events, can be ambiguous as it could be caused by plasma populations on more recently closed field lines where the plasma has not yet had a chance to isotropize (corresponding in the southward IMF paradigm to the plasma sheet boundary layer, and consistent with the Milan et al., 2005 interpretation), or it could be due to an injected population from the magnetopause which has mirrored at low altitudes (in the Shi et al., 2013 interpretation). However, in these three cases, the comparison with simultaneously observed properties of the plasma sheet (for Events 1 and 2) and spatial variation in the energy of the population (Event 3) provide strong additional supporting evidence in favor of the Milan et al. (2005) explanation.

Observations of transpolar arcs further aided our understanding of these energetic plasma populations. In the case of Event 2, in which conjugate arcs were observed, this confirmed that the plasma observed is on closed field lines (and that the northern and southern hemisphere are magnetically mapped [Milan et al., 2005]). The addition of SSUSI data in Event 2 revealed that there were in fact multiple sources of hot plasma embedded in the lobe. The significant spatial separation of the Cluster tetrahedron allowed us to utilize the differences in the location of the Cluster footprints. Figure 11 showed that these footprints intersected separate “strands” of plasma which extended poleward within the polar cap and which corresponded to multiple arcs. This shows for the first time that multiple instances of such hot plasma can be observed due to spatial effects (multiple structures, as inferred by Huang et al., 1987), and contrasts nicely with Event 1 in which multiple intermittent plasma populations occurred due to the same structure moving past the spacecraft multiple times (Fear et al., 2014).

## 5 Conclusion

In conclusion, all three events presented herein exhibited evidence of “hot” plasma embedded in the lobes. We observed comparable energies from direct observations of the plasma sheet in Events 1 and 2 and although there were no simultaneous plasma sheet observations available for Event 3, the energies of the plasma populations were consistent with the other two cases. All events analyzed in this study coincided with observations of transpolar arcs either in the Northern or Southern hemisphere, or both hemispheres simultaneously. Event 2 provided both observations of conjugacy which confirmed the plasma population is on closed field lines, and new insight into the formation of multiple hot plasma populations that can occur simultaneously but are separated by open field lines. In Event 1, a double loss cone was observed at discrete intervals as detailed by Fear et al. (2014); this was embedded within a period of bi‐directional pitch angle distributions, which were observed for the majority of the event. Similarly, bi‐directional distributions were observed for Events 2 and 3, with evidence of isotropization consistent with the evolution of plasma sheet structure as described by Walsh et al. (2013). The motion of the Cluster spacecraft for Events 1 and 2, as inferred from the mapped footprints, can be interpreted as being mainly parallel to the magnetic field. This was not the case for Event 3 in which the spacecraft had a significant poleward component of motion, moving onto higher latitude field lines. The decrease in energy seen when the Cluster spacecraft moved to field lines that map further poleward gives additional evidence to support that the changes in energy of the plasma are a result of plasma being observed at different latitudes within a closed field line structure within in the lobe.

This combination of observations allows us to conclude that the uncharacteristically energetic plasma observed in the lobes is consistent with the Milan et al. (2005) model, for which a wedge of closed flux in the otherwise open lobe regions results from tail reconnection under northward IMF conditions.

## Supporting information

Supporting Information S1

## Data Availability

The Cluster, Double Star, and IMAGE data are available through the Cluster Science Archive website https://csa.esac.esa.int/csa-web/. The SSUSI data are available from the SSUSI website https://ssusi.jhuapl.edu/. The Tsyganenko 96 model (Tsyganenko, 1996) and code used to trace the footprints of the Cluster spacecraft was accessed through a Python wrapper and is available open‐source through GitHub repository (Coxon & de Larquier, 2020). The Python packages used to conduct data analysis and visualisation were aacgmv2 (Shepherd, 2014), NumPy (Van Der Walt et al., 2011), and Matplotlib (Hunter, 2007).
