# Supplementary material for: Observations of Closed Magnetic Flux Embedded in the Lobes During Periods of Northward IMF
Source: J Geophys Res Space Phys. 2021 Jun 1;126(6):e2021JA029281. doi: 10.1029/2021JA029281 (PMC11578173; doi:10.1029/2021JA029281)
Supplement: Supplementary file 1 — Supporting Information S1 [file JGRA-126-0-s001.pdf]

AGU PUBLICATIONS

Journal of Geophysical Research

Supporting Information for

Observations of closed magnetic flux embedded in the lobes during periods of northward IMF

L. J. Fryer[1], R. C. Fear[1], J. C. Coxon[1], I. L. Gingell[1]

[1]School of Physics & Astronomy, University of Southampton, Southampton, UK.

Contents of this file

Text S1
Figures S1 to S3

Introduction

We have utilised the COmposition and DIstribution Function analyser (CODIF) instrument (Reme et al., 1997), which is on-board the Cluster suite, to analyse the ion components in more details than is available by Double Star. CODIF measured energy ranges up to 40keV/e for H^+, He^+, He^{++} and O^+ ions. We have chosen to utilise the moments provided by this instrument, more specifically the density, and the energy spectrograms for H^+ and O^+, to indicate the main contributing ion components within each of the Events and compare this to local plasma sheet conditions. To do this, we have chosen the closest time prior each Event in which Cluster was positioned within the plasma sheet. We have used the Cluster 3 spacecraft to compare the plasma populations observed in the three Events with the plasma sheet populations as this spacecraft contained the best coverage of data for all events.

Text S1.

Shi et al. (2013) discussed the relationship of the ratio of O^+ to H^+ density in their events. They argued that due to the fact the density ratio between these two components was low in their case study, this was evidence for direct entry from solar wind due to the lack of upwelling O^+ from the ionosphere. For Events 1 and 3, we observe similar ratios measuring around 0.2 for the times in which the most energetic plasma was present (panel S1j and S3j). For Event 2, the ratio was much lower, rarely measuring above 0.05 (panel S2j). We compare this to the ratios Cluster measured on the plasma sheet crossing preceding each event and can see that the ratio in the plasma sheet also varies, with values of order 0.2 (with majority of points in the range 0-0.4) for the plasma sheet crossing preceding Event 3, but closer to zero for the crossing preceding Events 1 and 2. However, the ion spectrograms for the plasma sheet crossings show the presence of O^+ at comparable energies to the plasma sheet H^+ ions (i.e above 10^4 eV) in all three cases (Figures S1c, S2c and S3c), consistent with the fact that O^+ can be present in the plasma sheet (Kistler et al, 2010). Clearly, the differential energy flux of the O^+ in the plasma sheet crossing preceding Events 1 and 2 is not significant enough to translate into a large enough density to influence the O^+/H^+ density ratio, but the range of the O^+/H^+ ratio observed in the three plasma sheet crossing shown here indicates that the values of the O^+/H^+ ratios observed in our three events is consistent with a plasma sheet origin, and hence the Milan et al. (2005) mechanism. Therefore, we take caution when inferring the mechanism for the presence of hot plasma embedded in the lobes from the density ratio of O^+ and H^+ components alone as the absence of O^+ does not rule out a plasma sheet origin, and indeed the fact that the populations are similar to the energies of the plasma sheet H^+ population is more consistent with the Milan et al. (2005) explanation. (NB This is contrary to the O^+ population observed between 16:00 and 17:00 UT in Event 1, which is more moderate and tightly confined in energy and is consistent with an upwelling population in the lobe.) We conclude that the O^+ and H^+ spectrograms in both the lobe and plasma sheet exhibit similar features. Consideration in particular of the energies of these populations (information on which is lost when considering density ratios alone) show that this O^+ population is more consistent with a plasma sheet origin (i.e the Milan et al. (2005) mechanism) than solar wind entry as was presented by Shi et al. (2013).

References.

Kistler, L. M., Mouikis, C. G., Klecker, B., and Dandouras, I., "Cusp as a source for oxygen in the plasma sheet during geomagnetic storms", Journal of Geophysical Research (Space Physics), vol. 115, no. A3, 2010. doi:10.1029/2009JA014838.

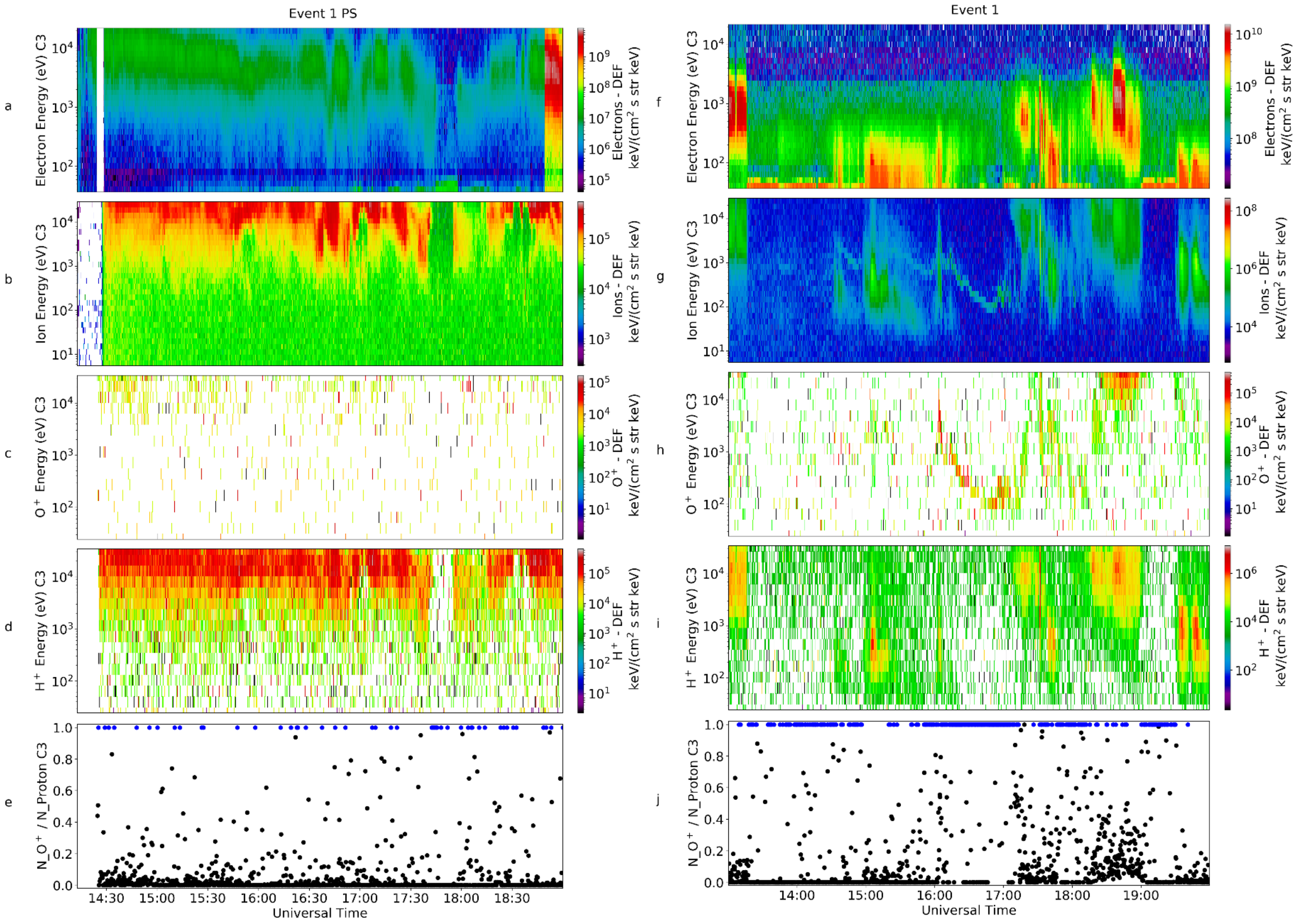

Figure S1. Data from the Cluster 3 spacecraft over the period of 14:15 UT – 19:00 UT on the 14/09/2005 when it was located within the plasma sheet on the left and the full period of Event 1 (13:00 UT – 20:00 UT) on the right. Panel a, shows spectrograms of the energy of the electrons by the PEACE instrument and b, the energy of the ion population measured by HIA. Panels c and d show the energy of the O^+ and H^+ ion components measured by CODIF. Panel e is the ratio of the mass density of O^+ and H^+, with the blue points representing any ratio above 1. Panels f-j show the same parameters as panels a-e but for when Cluster was in the lobe.

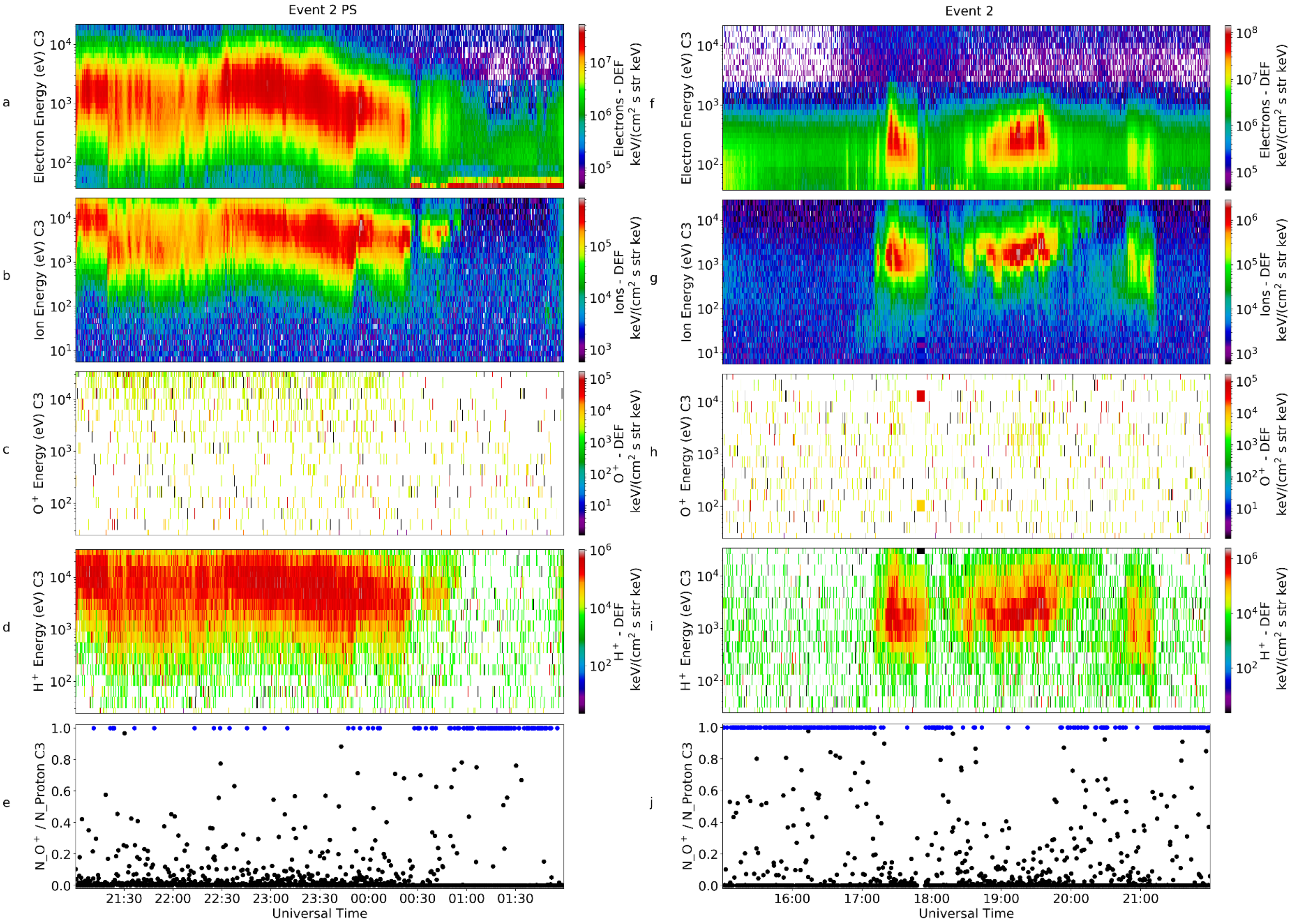

Figure S2. Data from the Cluster 3 spacecraft over the period of 21:00 UT on the 28/09/2005 – 02:00 UT on the 29/09/2005 when it was located within the plasma sheet on the left and the full period of Event 2 (15:00 UT – 22:00 UT 30/09/2005) on the right. This figure has the same format at Figure S1.

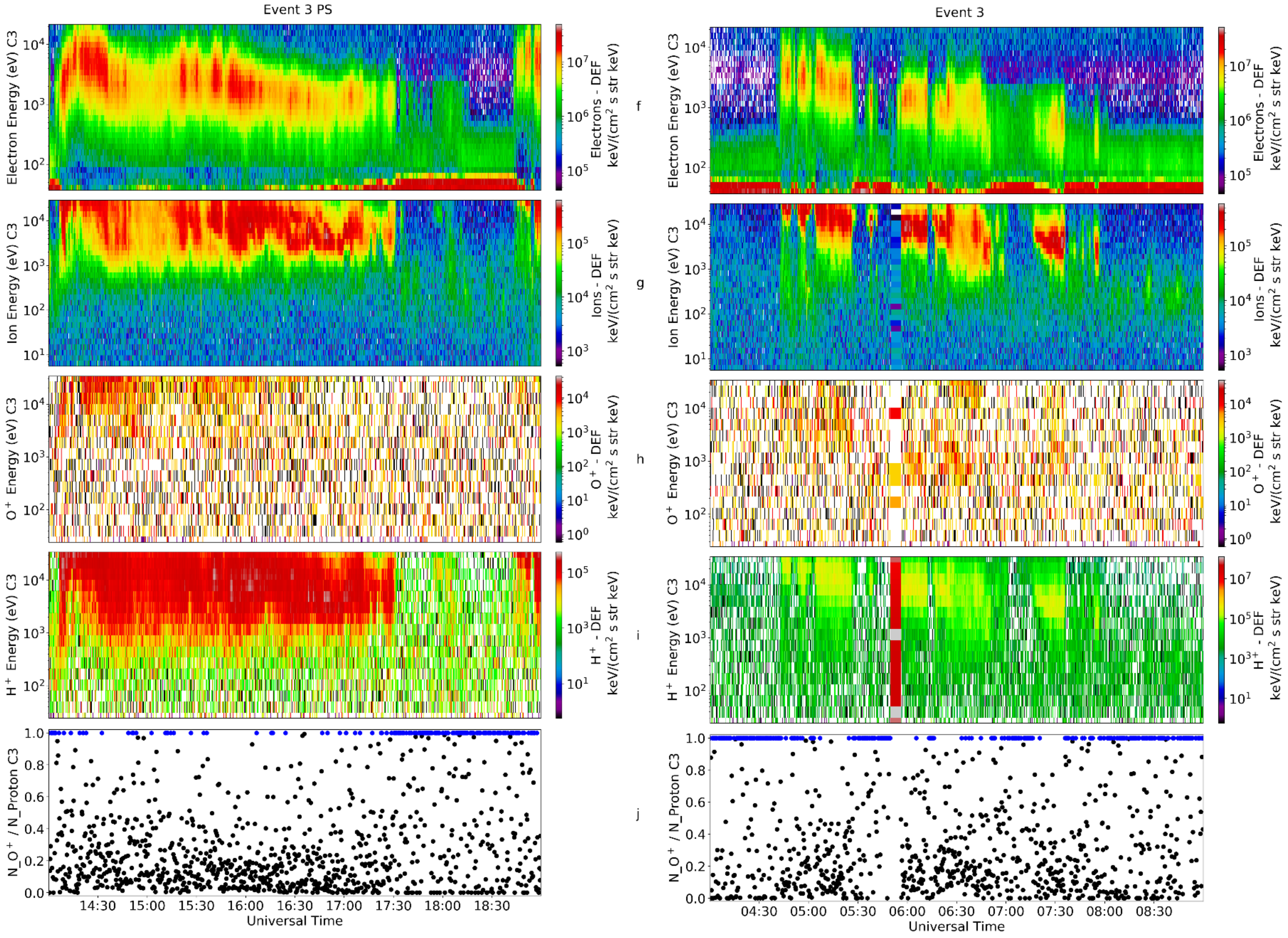

Figure S3. Data from the Cluster 3 spacecraft over the period of 14:00 UT – 19:00 UT on the 10/09/2003 when it was located within the plasma sheet on the left and the full period of Event 3 (04:00 UT – 09:00 UT 11/09/2003) on the right. This figure has the same format at Figure S1.
